# The quality of malaria case management in different transmission settings in Tanzania mainland, 2017–2018

**DOI:** 10.1371/journal.pgph.0002318

**Published:** 2023-08-21

**Authors:** Ally Kassim Hussein, Donath Tarimo, Erik J. Reaves, Frank Chacky, Ahmed Mohamed Abade, Charles Dismas Mwalimu, Ally Mohamed, Ahlam Nasser, Rogath Saika Kishimba

**Affiliations:** 1 Tanzania Field Epidemiology and Laboratory Training Program, Dar es Salaam, United Republic of Tanzania; 2 Muhimbili University of Health and Allied Sciences, Dar es Salaam, United Republic of Tanzania; 3 U.S. President’s Malaria Initiative, U.S. Centers for Disease Control and Prevention, Dar es Salaam, United Republic of Tanzania; 4 National Malaria Control Program, Dodoma, United Republic of Tanzania; 5 Ministry of Health, Dodoma, United Republic of Tanzania; Tulane University School of Public Health and Tropical Medicine, UNITED STATES

## Abstract

Tanzania is undergoing an epidemiological transition for malaria transmission with some areas of the country having <10% (hypoendemic) and other areas 10% - 50% malaria prevalence (mesoendemic). It is not known whether there is a difference in the quality of malaria case management based on endemicity in Tanzania mainland. We examined the influence of endemicity on the quality of malaria case management at health facilities. We conducted a cross-sectional analysis of 1713 health facilities in Tanzania mainland, using data collected by the National Malaria Control Program through an assessment tool to evaluate quality of malaria case management. The data was gathered from September 2017 to December 2018. Using standard quality factors, mean scores from facilities in the different endemicity regions were compared by a Student’s t-test. Simple and multiple linear regression analyses were performed to determine the association between facility performance (score) and endemicity (mesoendemic vs. hypoendemic). Facilities in mesoendemic regions scored higher than those in hypoendemic regions on the overall quality of services [difference in mean scores ($\bar{d}$) = 2.52; (95% Confidence Interval (CI) 1.12, 3.91)], site readiness [$\bar{d}$ = 2.97; (95% CI 1.30, 4.61)], availability of malaria reference materials [$\bar{d}$ = 4.91; (95% CI 2.05, 7.76)], availability of Health Management Information System tools [$\bar{d}$ = 5.86; (95% CI 3.80, 7.92)] and patient satisfaction [$\bar{d}$ = 6.61; (95% CI 3.75, 9.48)]. Predictors associated with lower facility scores included; being located in a hypoendemic region [β: -2.49; (95% CI -3.83, -1.15)] and urban area [β: -3.84; (95% CI -5.60, -2.08)]. These findings highlight the differences in quality of malaria case management based on endemicity, but there is still a need to target improvement efforts in underperforming facilities, regardless of endemicity.

## Introduction

In 2021, nearly half of the world’s population was at risk of malaria [1], making it the most important human public health parasitic disease. A documented new global strategy for malaria control dates back to 1993 [2], and after two decades of scaling up effective control tools to reduce morbidity and transmission, major achievements have been recorded [1, 3]. In 2021, approximately 247 million cases and 619,000 deaths were reported globally. The majority of the morbidity (95%) and mortality (96%) occurred in sub-Saharan Africa [1].

A major decline in malaria prevalence has been witnessed in Tanzania. Malaria prevalence among children under the age of five has declined more than 50%, from 18.1% in 2007 to 7.9% in 2022. Some regions such as Arusha and Manyara have reached a prevalence of <1%, while in other regions prevalence has remained high at ≥10% [4–6]. The global malaria strategy emphasizes the need for stratification of areas to effectively target malaria interventions. This stratification allows for a targeted approach in developing specific case management strategies that align with the unique characteristics and needs of the different areas [7]. However, the majority (96%) of the population in Tanzania mainland is still at risk for malaria infection [4, 6].

In Tanzania mainland, 80% of all patients visiting the health facilities are attended as outpatients [8, 9]. Prompt diagnosis and treatment with an effective antimalarial reduces morbidity and prevents mortality [10] and is one of the core interventions in controlling malaria [7]. Appropriate management of cases might also reduce transmission by reducing the human parasite reservoir and prevent the emergence of drug resistance [10–13]. Furthermore, improving case management may also contribute to improved treatment of non-malarial febrile illnesses, which are often misdiagnosed and treated presumptively as malaria [14, 15]. As countries progress toward elimination, malaria interventions should focus on rigorous case management and transmission control [16]. While there are fewer malaria fevers in these settings, patients still consult health facilities for non-malarial fevers [15]. Since fever is the entry point for malaria case management [17], the higher prevalence of and priority given to management of non-malaria fevers might compromise the quality of malaria case management [18].

The quality of malaria case management provided in health facilities in regions progressing toward elimination has not been evaluated in Tanzania mainland [15]. This study aimed to compare the quality of malaria case management provided at outpatient departments in hypoendemic versus mesoendemic settings.

The information generated from comparing the quality of malaria case management in these different settings will facilitate targeting strategies to improve the quality of services in lower performing health facilities. Thus, will ensure that all individuals receive the best possible care for malaria, regardless of where they live.

## Methods

### Malaria service and data quality improvement (MSDQI) package

The MSDQI package is part of a national strategy for monitoring and improving malaria services provided in health facilities and assessing the quality of routinely collected malaria data reported from health facilities [19]. The package was developed by the National Malaria Control Program (NMCP) and development partners in line with the National Health and Social Welfare Quality Improvement Strategic Plan [20], the Tanzania Quality Improvement Framework in Health Care [21] and Situation Analysis of Quality Improvement in Health Care [22].

The MSDQI package consists of seven modules, each accompanied by a corresponding checklist. These modules assess various areas, including the outpatient department (OPD), malaria Rapid Diagnostic Test (mRDT), antenatal care (ANC) clinic, inpatient department (IPD) for severe malaria, logistics and supply chain for malaria commodities, malaria microscopy, and a Data Quality Audit (DQA) conducted in health facilities [19].

Trained Council Health Management Team (CHMT) members perform health facility supportive supervision visits, including MSDQI package supervision, at least once per quarter. The NMCP trained seven CHMT staff in every council on performing supervision visits using the MSDQI package. The trainings were conducted for a period of six days during the rollout of MSDQI [19].

MSDQI package supervision visits began in 2017. Data was collected via two means, either by electronic-based checklists on android smartphones or tablets, known as the Electronic Data System (EDS), or by paper-based checklists. Supervision visits documented by paper-based checklists were later entered into the EDS [23]. Supervisors for each module conducted supervision visits, completed the checklists, calculated quality performance scores for supervision visits conducted with paper-based checklists, and completed the quality improvement plan to address deficiencies identified through the MSDQI package during the supervision visit. Upon completion of the supervision visit, the supervisors entered the scores into the District Health Information Software 2 (DHIS2) system. The MSDQI checklists completed in the EDS were capable of automatically generating the quality scores and uploading the data to DHIS2. The MSDQI data and score results could be visualized in and retrieved from the ‘Malaria Dashboard’ user interface in DHIS2 [19].

### Study setting and design

We conducted an analytical cross-sectional study to compare the quality of health-facility based malaria case management in malaria hypoendemic versus mesoendemic regions in Tanzania mainland.

Tanzania mainland is divided into 26 administrative Regions which are further subdivided into Councils. There are a total of 184 Councils. Councils are categorised according to population settings with 137 being rural and 47 being urban. There is one type of rural Council: a District Council. There are three types of urban Councils: Town, Municipal and City Councils [24].

This study only uses data from the MSDQI OPD checklist, which is one of the seven modules in the MSDQI package. Available MSDQI scores from health facility OPD checklists completed during supportive supervision visits conducted between September 1, 2017 and December 31, 2018 were retrieved from DHIS2 for analysis.

### Variables

Regions were classified as hypoendemic when malaria prevalence ranged between 0 - <10% and mesoendemic when prevalence ranged between 10–50% among children (6–59 months) [25]; prevalence was estimated using malaria infection detected by SD BIOLINE Malaria Ag P.f/Pan rapid malaria tests (Standard Diagnostics Inc., Republic of Korea) during the 2017 Tanzania Malaria Indicator Survey [26]. There were no regions classified as hyperendemic (>50–75%) or holoendemic (>75%) during the survey.

Malaria case management services are provided across all types of health facilities in Tanzania. A dispensary, located at village level, offers services on an outpatient basis. Health centres, at ward level, are the first referral centres for dispensaries, and also provide inpatient and diagnostic services. Hospitals are located at council and regional levels to serve as referral centres with a full complement of health services [27]. Health facilities were assigned to their respective urban or rural setting category based on the council they were located in. Implementing partners are nongovernmental organizations that provide financial and technical support to improve the provision of malaria case management services across all facility types, often with an emphasis in regions with the highest burden of malaria.

Tanzania’s rainfall pattern is heterogeneous across the country due to effects of topography, winds, humidity, and tropical circulation [28]. Regional rainfall seasonality at the time (month) of health facility assessment was categorised into either dry or wet (rainy) using a cut off value of 100 mm.

Information describing the quality of malaria case management was organised by sub-factors grouped into three broad factors: site readiness, clinical management, and outcome according to the Donabedian model “Table 1” [29]. For each health facility, an overall score was calculated as the mean of the sub-factor scores for each of the broad factors.

**Table 1:** Factors assessed for the quality of malaria case management in the malaria service and data quality improvement outpatient department checklist.

Factors	Sub-Factors	Explanation
Site readiness	Human resource	Availability of the minimum number of staff required at the OPD
	Staff training	Availability of clinical staff at the OPD who have received malaria case management training
	Malaria reference materials	Availability of malaria reference materials, e.g., national treatment guidelines, treatment charts, etc.
	Essential equipment	Availability and functionality of equipment required for managing a febrile patient, e.g., thermometers, weighing scale, etc.
	Health Management Information System (HMIS) tools	Availability of HMIS book 5 (register, tally sheet, and summary forms)
	Overall site readiness	A composite score, a mean of all site readiness sub-factor scores (above)
Clinical management	History taking	The clinician’s history taking skills while attending a febrile patient, e.g., age, duration of fever, diarrhoea, convulsions, etc.
	Physical examination	The clinician’s physical examination skills performed, e.g., signs of anaemia, neck exam, measurement of temperature, etc.
	Malaria testing	Whether the clinician orders or conducts a test for malaria, and whether he/she waits for the results before prescribing medication or making the final diagnosis.
	Malaria diagnosis	Whether the clinician made a clinical or confirmed diagnosis, and whether the test results were interpreted correctly, e.g., negative malaria Rapid Diagnostic Test with a non-malaria cause of fever.
	Malaria treatment	Whether the patient with a positive malaria test was prescribed the right dose of an antimalarial. Or not prescribed antimalarial if a negative test.
	Patient counselling	Whether the patient was counselled on how to properly take antimalarials at home, when to return, use a bed net, etc.
	Overall clinical management	A composite score, a mean of all clinical management sub-factor scores (above)
Outcome	Patient satisfaction	Patient’s level of satisfaction with the services received
Overall quality of case management		A composite score, a mean of the site readiness, clinical management, and outcome factor scores

Site readiness describes the context in which care is delivered. Information on subfactors in site readiness was obtained by interviews with health facility staff and observations.

Clinical management include all transactions made between the patients and health service providers from the time of history taking up to when the patient exits the facility. Information on subfactors in clinical management were obtained by observing the management of four febrile patients, including two under five years old & two over five years old.

Outcome factors refer to the effects of healthcare on the health status of patients and populations. This includes client satisfaction and changes in malaria morbidity in the region. Information on client satisfaction was obtained by exit interviews of two febrile patients. In this study, the outcome factor of malaria endemicity was used to categorize the facilities into the comparison groups i.e. those in Malaria Hypoendemic or Mesoendemic settings.

All sub-factors listed in “Table 1” are numerical variables and range from 0–100. The scores were awarded based on the performance against the standards set in the MSDQI OPD checklist. The scores were then graded into 3 categories as follows: 75–100 (Good), 50 - <75 (Average) and 0-<50 (Poor). The full MSDQI OPD checklist has been added as “S1 Checklist”.

### Data retrieval

MSDQI supervision data from the health facilities were retrieved from DHIS2 via pivot table application and downloaded as comma-separated values files. Information on health facility type, location, level, and ownership were obtained from the Health Facility registry. Regional rainfall seasonality patterns were obtained from the Tanzania Meteorological Agency Map room [30]. Data were merged in Microsoft Excel 2013 (Microsoft Office Professional Plus 2013, Seattle WA). Data used in the study has been added as “S1 Data”.

### Data analysis

Merged data were analysed using STATA version 15.1 (STATA Corp, Texas, USA). Descriptive statistics were summarised by frequencies, percentages, and means where appropriate. A Chi-square test was used to compare the distribution of health facilities between the MSDQI scores and endemicity. The means of scores for the facilities in malaria mesoendemic and hypoendemic settings were compared using a Student’s t-test to determine whether there was a difference in the site readiness, clinical management, and outcome factors of the quality of malaria case management. A simple linear regression analysis followed by a standard multiple linear regression analysis were performed to determine the association between the quality of malaria case management (dependent) and other variables (independent) i.e., malaria endemicity, health facility location (urban or rural), facility type according to the level of care provided (hospitals, health centers, or dispensaries), facility ownership (public, private for profit, private Non-Governmental Organizations or Faith-Based Organizations), the season when the facility was surveyed (wet or dry), and whether the region has implementing partners supporting malaria case management (yes or no). Independent variables were added into the model using forward selection. To address the issue of multicollinearity, a threshold of variance inflation factor (VIF) equal to 5 was chosen. Variables with VIF values exceeding this threshold were considered to have a high level of collinearity and were subsequently removed from the regression model to ensure the stability and reliability of the estimated coefficients. The linear regression analyses were also performed for site readiness, clinical management and patient satisfaction. P-values <0.05 were considered statistically significant. Maps were drawn using Q-GIS Desktop 2.18.13 (QGIS Development Team, Switzerland). All shapefiles used are from openly available sources. (https://www.nbs.go.tz/index.php/en/census-surveys/gis/385-2012-phc-shapefiles-level-one-and-two). The shapefiles were made based on the 2012 population and housing census.

### Ethical consideration

Ethical clearance was obtained from the Institutional Ethical Review Board of Muhimbili University of Health and Allied Sciences; Reference Number DA.287/298/01A. Additional permission to conduct the study and use the extracted MSDQI programmatic data was obtained from the National Malaria Control Program. None of the data included individual or patient identifying information.

## Results

**Fig 1:**
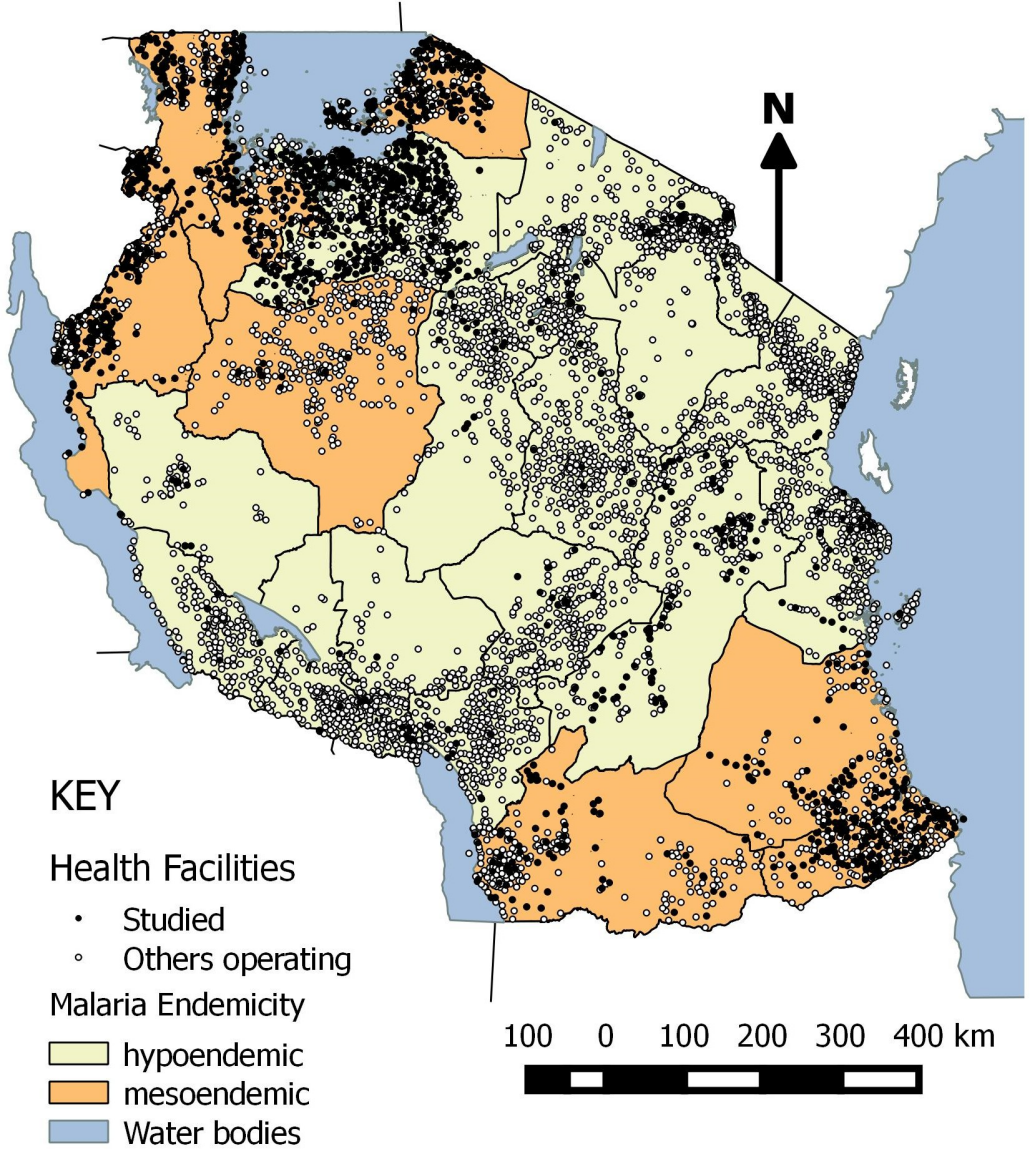
Distribution of health facilities by region and malaria endemicity.

Between the 1^st^ of September 2017 and 31^st^ of December 2018, a total of 1713 health facilities (21.3% of the overall 8033 operating facilities located across all 26 regions) in Tanzania mainland reported data into DHIS2 which included factors of quality case management assessed through the MSDQI OPD checklists conducted by CHMTs. ""Fig 1,"" shows the distribution of health facilities reporting MSDQI data into DHIS2 during the study period by region and malaria endemicity. MSDQI data was available from 992 (57.9%) facilities reporting from regions in the Lake and Southern Zones of Tanzania. Simiyu region had the highest MSDQI OPD coverage at 74.3%, while Dar es Salaam had the lowest coverage at 0.5% “S1 Table”.

The shapefile used is from an openly available source. (https://www.nbs.go.tz/index.php/en/census-surveys/gis/385-2012-phc-shapefiles-level-one-and-two). The shapefile was made based on the 2012 population and housing census.

Out of the 1713 health facilities, more than half (57.6%) were located in malaria mesoendemic settings.

Thirteen hundred and ninety-six (81.5%) of the health facilities were dispensaries. The distribution of health facilities by managing authority and location were similar between hypoendemic and mesoendemic settings (p-values 0.466 and 0.742, respectively). Nine hundred and seventy-eight (99.1%) of the health facilities in the mesoendemic settings had *implementing partners* supporting malaria case management. Nine hundred and forty-two (55.0%) of the facilities were evaluated during the dry season “Table 2”.

**Table 2:** Health facility characteristics by malaria endemicity.

Variables	Categories	Mesoendemic (n = 987) n (%)	Hypoendemic (n = 726) n (%)	Total (n = 1713) n (%)	P-value
Health facility level	Hospital	42 (4.3)	49 (6.8)	91 (5.3)	0.008
	Health Centre	117 (11.8)	109 (15.0)	226 (13.2)	
	Dispensary	828 (83.9)	568 (78.2)	1396 (81.5)	
Health facility managing authority	Public	820 (83.1)	605 (83.3)	1425 (83.2)	0.466
	NGO/FBO	83 (8.4)	69 (9.5)	152 (8.9)	
	Private for profit	84 (8.5)	52 (7.2)	136 (7.9)	
Health facility location	Urban	187 (18.9)	133 (18.3)	320 (18.7)	0.742
	Rural	800 (81.1)	593 (81.7)	1393 (81.3)	
Season surveyed	Wet (rainy)	505 (51.2)	266 (36.6)	771 (45.0)	<0.001
	Dry	482 (48.8)	460 (63.4)	942 (55.0)	
Partner support	Yes	978 (99.1)	562 (77.4)	1540 (89.9)	<0.001
	No	9 (0.9)	164 (22.6)	173 (10.1)	

NGO = Non-Governmental Organization; FBO = Faith-Based Organization

The distribution of all operating facilities in Tanzania mainland by endemicity setting are presented in “S2 Table”.

Nearly all of the health facilities scored Good or Average in the overall quality of case management (95.5%) and site readiness (95.8%). Approximately one third 553 (32.3%) of the facilities scored poorly in clinical management. A higher proportion of facilities, irrespective of level or type, in the mesoendemic regions had higher good scores compared to those in the hypoendemic regions in all categories except for clinical management “Table 3”.

**Table 3:** Health facility distribution based on MSDQI scores by endemicity.

Factor	Score	Mesoendemic (n = 987)	Hypoendemic (n = 726)	Total (n = 1713)	P-value
		n (%)	n (%)	n (%)	
Overall quality of case management	Good	596 (60.4)	404 (55.7)	1000 (58.4)	<0.001
	Average	370 (37.5)	266 (36.6)	636 (37.1)	
	Poor	21 (2.1)	56 (7.7)	77 (4.5)	
Site readiness	Good	691 (70.0)	460 (63.4)	1151 (67.2)	<0.001
	Average	270 (27.4)	220 (30.3)	490 (28.6)	
	Poor	26 (2.6)	46 (6.3)	72 (4.2)	
Clinical management	Good	290 (29.4)	271 (37.3)	561 (32.7)	0.002
	Average	366 (37.1)	233 (32.1)	599 (35.0)	
	Poor	331 (33.5)	222 (30.6)	553 (32.3)	
Outcome (patient satisfaction)	Good	702 (71.1)	469 (64.6)	1171 (68.4)	<0.001
	Average	184 (18.7)	129 (17.8)	313 (18.3)	
	Poor	101 (10.2)	128 (17.6)	229 (13.3)	

Scores graded as Good (75–100), Average (50 - <75), and Poor (0 - <50)

S1–S4 Figs illustrate the facilities performances graphically.

Facilities in the malaria mesoendemic regions had a higher mean *Overall quality of case management* score compared to those in hypoendemic regions [Difference in mean scores ($\bar{d}$) = 2.52; (95% CI 1.13, 3.91)].

Facilities in the malaria mesoendemic regions scored higher in the availability of malaria reference materials [$\bar{d}$ = 4.91; (95% CI 2.05, 7.76)], availability of HMIS tools [$\bar{d}$ = 5.86; (95% CI 3.80, 7.92)], and the *Site readiness* score [$\bar{d}$ = 2.97; (95% CI 1.31, 4.61)].

Facilities in the malaria mesoendemic regions had higher mean scores of the main *Outcome*, i.e., patient satisfaction [$\bar{d}$ = 6.61; (95% CI 3.75, 9.48)] “Table 4”.

**Table 4:** Difference in mean scores between malaria hypoendemic and mesoendemic regions.

Factors	Mesoendemic Mean (SD)	Hypoendemic Mean (SD)	Difference in Means (95% CI)	P-value
***Overall quality of case management***	76.72 (11.74)	74.2 (16.26)	2.52 (1.13, 3.91)	<0.001
***Site readiness***	80.17 (13.48)	77.2 (19.59)	2.97 (1.31, 4.61)	<0.001
Human resources	73.31 (29.53)	75.4 (30.17)	-2.09 (-4.95, 0.77)	0.152
Staff training	57.50 (33.10)	57.64 (34.0)	-0.14 (-3.43, 3.15)	0.936
Reference materials	67.72 (27.60)	62.81 (31.33)	4.91 (2.05, 7.76)	<0.001
Essential equipment	79.13 (18.56)	80.30 (22.84)	-1.17 (-3.19, 0.87)	0.246
HMIS tools	94.11 (15.55)	88.26 (24.97)	5.86 (3.80, 7.92)	<0.001
***Clinical management***	58.74 (25.31)	59.96 (28.17)	-1.22 (-3.18, 1.36)	0.346
History taking	49.51 (25.42)	50.77 (29.08)	-1.26 (-3.90, 1.35)	0.341
Physical examination	43.80 (28.23)	46.19 (31.30)	-2.39 (-5.26, 0.49)	0.099
Malaria testing	69.81 (29.68)	69.04 (34.19)	0.77 (-2.34, 3.87)	0.621
Malaria diagnosis	74.16 (31.15)	76.04 (34.68)	-1.88 (-5.07, 1.31)	0.239
Malaria treatment	67.76 (31.21)	69.78 (32.85)	-2.02 (-5.10, 1.06)	0.196
Patient counselling	47.38 (31.62)	47.94 (33.44)	-0.56 (-3.70, 2.56)	0.719
***Outcome***				
Patient satisfaction	76.75 (26.25)	70.14 (32.28)	6.61 (3.75, 9.48)	<0.001

Facilities in regions with implementing partner support irrespective of endemicity setting had higher mean overall quality of case management, site readiness, clinical management, and patient satisfaction scores compared to those without partner support (p-values <0.001) “Table 5”.

**Table 5:** Difference in mean scores between facilities with (n = 1540) and without (n = 173) case management implementing partners.

Factor	With Partner Support Mean (SD)	Without Partner Support Mean (SD)	Difference in Means (95% CI)	P-value
Overall quality of case management	77.02 (11.70)	63.44 (22.98)	13.58 (10.08, 17.07)	<0.001
Site readiness	80.19 (14.40)	67.47 (26.03)	12.72 (8.75, 16.69)	<0.001
Clinical management	60.35 (25.81)	49.52 (30.86)	10.82 (6.02, 15.63)	<0.001
Patient satisfaction	75.61 (27.38)	59.11 (38.68)	16.50 (10.54, 22.46)	<0.001

Facilities with implementing partner support in malaria mesoendemic regions scored lower in clinical management [$\bar{d}$ = -3.75; (95% CI -6.47, -1.03)] compared to those in hypoendemic regions. However, they scored higher in patient satisfaction [$\bar{d}$ = 4.12; (95% CI 1.17, 7.07)]. There were no differences in the site readiness or overall quality of case management scores between the two endemicity settings (p-value = 0.994 and 0.772, respectively) “Table 6”.

**Table 6:** Difference of mean scores between malaria hypoendemic (n = 562) and mesoendemic (n = 978) regions with case management implementing partners.

Factor	Mesoendemic Mean (SD)	Hypoendemic Mean (SD)	Difference in Means (95% CI)	P-value
Overall quality of case management	76.96 (11.39)	77.14 (12.23)	-0.18 (-1.41, 1.06)	0.772
Site readiness	80.19 (13.42)	80.19 (15.98)	0.01 (-1.56, 1.57)	0.994
Clinical management	58.98 (25.16)	62.73 (26.77)	-3.75 (-6.47, -1.03)	0.006
Patient satisfaction	77.11 (25.78)	72.99 (29.79)	4.12 (1.17, 7.07)	0.004

A Significant Regression equation was found with an R^2^ of 0.018 on multiple linear regression analysis. Independent predictors of the *Overall Quality of Case Management* score that were found to be statistically significant included malaria endemicity (mesoendemic vs. hypoendemic) [β: -2.49; (95% CI -3.83, -1.15)], health facility location (rural vs. urban) [β: -3.84; (95% CI -5.60, -2.08)]. Partner support was not included in the final model because of collinearity with endemicity. *Overall Quality of Case Management* scores in health facilities located in hypoendemic regions were 2.49 points lower compared to those in mesoendemic regions. Those located in urban areas were 3.84 points lower compared to those in rural areas “Table 7”.

**Table 7:** Linear regression analysis results of overall quality of case management scores.

Variable	Unadjusted		Adjusted (R^2^: 0.018) p = <0.001	
	Estimated β coefficient (95% CI)	P-value	Estimated β coefficient (95% CI)	P-value
***Endemicity***				
Mesoendemic	Ref		Ref	
Hypoendemic	-2.52 (-3.85, -1.19)	<0.001	-2.49 (-3.83, -1.15)	<0.001
***Facility level***				
Dispensary	Ref		Ref	
Health Centre	-1.00 (-2.96, 0.95)	0.313	-0.61 (-2.56, 1.34)	0.541
Hospital	-2.33 (-5.28, 0.61)	0.121	-1.16 (-4.24, 1.92)	0.460
***Authority***				
Public	Ref		Ref	
NGO/FBO	-1.31 (-3.63, 1.02)	0.270	-0.75 (-3.16, 1.66)	0.541
Private	-2.17 (-4.61, 0.27)	0.082	-0.72 (-3.26, 1.82)	0.578
***Location***				
Rural	Ref		Ref	
Urban	-4.04 (-5.72, -2.37)	<0.001	-3.84 (-5.60, -2.08)	<0.001
***Season surveyed***				
Dry	Ref		Ref	
Wet	0.39 (-0.92, 1.72)	0.554	0.05 (-1.28, 1.38)	0.945
***Partner support***				
No	Ref			
Yes	13.58 (11.49, 15.67)	<0.001		

NGO = Non-Governmental Organisation; FBO = Faith-Based Organisation

Significant multiple linear regression results were found for predicting site readiness and client satisfaction scores. In facilities located in Hypoendemic regions, site readiness and client satisfaction scores were found to be 2.99 points and 6.99 points lower, respectively, compared to those in mesoendemic regions. The multiple linear regression results for predicting client satisfaction scores were not significant “Tables 8–10”.

**Table 8:** Linear regression analysis results for site readiness scores.

Variable	Unadjusted		Adjusted (R^2^: 0.01) p = <0.001	
	Estimated β coefficient (95% CI)	P-value	Estimated β coefficient (95% CI)	P-value
***Endemicity***				
Mesoendemic	Ref		Ref	
Hypoendemic	-2.96 (-4.53, -1.39)	<0.001	-2.99 (-4.58, -1.41)	<0.001
***Facility level***				
Dispensary	Ref		Ref	
Health Centre	-2.66 (-4.96, -0.35)	0.024	-2.27 (-4.59, 0.04)	0.054
Hospital	-3.54 (-7.02, -0.07)	0.046	-2.78 (-6.43, 0.87)	0.136
***Authority***				
Public	Ref		Ref	
NGO/FBO	-1.31 (-4.05, 1.43)	0.349	-0.32 (-3.18, 2.53)	0.827
Private	-2.44 (-5.32, 1.43)	0.098	-1.93 (-4.94, 1.08)	0.209
***Location***				
Rural	Ref		Ref	
Urban	-2.62 (-4.61, -0.63)	0.010	-2.07 (-4.15, 0.01)	0.051
***Season surveyed***				
Dry	Ref		Ref	
Wet	-0.61 (-2.17, 0.95)	0.442	-0.97 (-2.55, 0.60)	0.226
***Partner support***				
No	Ref			
Yes	12.72 (10.21, 15.22)	<0.001		

NGO = Non-Governmental Organisation; FBO = Faith-Based Organisation

**Table 9:** Linear regression analysis results for clinical management scores.

Variable	Unadjusted		Adjusted (R^2^: 0.0001) p = 0.169	
	Estimated β coefficient (95% CI)	P-value	Estimated β coefficient (95% CI)	P-value
***Endemicity***				
Mesoendemic	Ref		Ref	
Hypoendemic	1.22 (-1.32, 3.77)	0.346	0.76 (-1.82, 3.35)	0.562
***Facility level***				
Dispensary	Ref		Ref	
Health Centre	0.97 (-2.76, 4.71)	0.609	1.29 (-2.47, 5.06)	0.501
Hospital	0.57 (-5.07, 6.21)	0.843	0.89 (-5.05, 6.84)	0.769
***Authority***				
Public	Ref		Ref	
NGO/FBO	0.33 (-4.11, 4.78)	0.884	0.28 (-4.38, 4.93)	0.907
Private	-0.63 (-5.31, 4.05)	0.792	0.28 (-4.61, 5.18)	0.909
***Location***				
Rural	Ref		Ref	
Urban	-2.26 (-5.49, 0.97)	0.170	-2.42 (-5.82, 0.97)	0.161
***Season surveyed***				
Dry	Ref		Ref	
Wet	-2.66 (-5.18, -0.13)	0.039	-2.63 (-5.20, -0.06)	0.045
***Partner support***				
No	Ref			
Yes	10.82 (6.67, 14.97)	<0.001		

NGO = Non-Governmental Organisation; FBO = Faith-Based Organisation

**Table 10:** Linear regression analysis results for patient satisfaction scores.

Variable	Unadjusted		Adjusted (R^2^: 0.01) p = <0.001	
	Estimated β coefficient (95% CI)	P-value	Estimated β coefficient (95% CI)	P-value
***Endemicity***				
Mesoendemic	Ref		Ref	
Hypoendemic	-6.61 (-9.39, -3.83)	<0.001	-6.68 (-9.50, -3.86)	<0.001
***Facility level***				
Dispensary	Ref		Ref	
Health Centre	-1.76 (-5.86, 2.32)	0.398	-1.16 (-5.28, 2.94)	0.577
Hospital	-4.76 (-10.94, 1.41)	0.131	-3.98 (-10.48, 2.51)	0.229
***Authority***				
Public	Ref		Ref	
NGO/FBO	-4.37 (-5.31, 4.44)	0.860	0.87 (-4.21, 5.95)	0.736
Private	-1.77 (-6.91, 3.35)	0.497	-1.54 (-6.88, 3.80)	0.571
***Location***				
Rural	Ref		Ref	
Urban	-2.21 (-5.75, 1.33)	0.221	-1.75 (-5.45, 1.94)	0.352
***Season surveyed***				
Dry	Ref		Ref	
Wet	-0.19 (-2.97, 2.57)	0.888	-1.14 (-3.94, 1.66)	0.424
***Partner support***				
No	Ref			
Yes	16.50 (11.99, 21.02)	<0.001		

NGO = Non-Governmental Organisation; FBO = Faith-Based Organisation

## Discussion

In this study, we found that health facilities located in malaria hypoendemic regions scored lower than those in mesoendemic regions in terms of the overall quality of services provided for malaria, overall site readiness to provide care, availability of malaria reference materials, availability of HMIS tools, and patient satisfaction. However, there was no difference in the clinical management factors between facilities in hypoendemic compared to mesoendemic regions. Facilities located in urban areas were also found to score lower than those in rural areas, and those without implementing partner support scored lower during the MSDQI assessments. Nevertheless, this comparison may help decision makers to identify areas that need to be targeted for improvement in future analyses, especially considering that supervision is provided to only a limited number of facilities. Possible areas to focus improvements based on our analysis are site readiness, especially staff training, and clinical management.

### Site readiness

Facilities located in malaria mesoendemic regions were found to have higher readiness scores compared to those in hypoendemic regions; this is consistent with findings from a study among 826 health facilities in Kenya, Namibia, and Senegal [31]. This might be explained by having a higher proportion of facilities located in rural settings included in the analytical samples. Malaria has traditionally been a rural disease; the higher malaria burden may lead to improved facility readiness in mesoendemic settings [31, 32].

The type of health facility did not influence the readiness to provide care for malaria patients. This contradicts previous findings from studies conducted in low and middle-income countries, including Tanzania [33], which showed there were differences in readiness by the type of the facility. This might be due to having a different composition in the types of facilities; more than three quarters (81.5%) of the facilities involved in this study were dispensaries (i.e., lowest facility level). Another possible reason might be that malaria is among the most common diagnoses attended in health facilities in the country and the standards for operating facilities require malaria services to be delivered in facilities at all levels; this might explain the high level of facility readiness in our analysis regardless of facility type.

Facilities in mesoendemic regions were found to have a higher availability of reference materials. A possible explanation for the observed difference could be due to the presence of implementing partners providing case management support, which includes printing and distribution of reference materials to health facilities. A higher proportion of facilities in the mesoendemic regions had implementing partner support compared to those in hypoendemic regions. Another reason could be due to the number of reference materials used in the calculation of the indicator. This indicator was comprised of five different reference materials, some of which might not be frequently used in facilities in hypoendemic regions, resulting in lower scores during the evaluation.

In both endemicity settings, health facilities scored best in the availability of HMIS tools compared to other factors. This could be explained by the recent health systems strengthening efforts for improving the HMIS in Tanzania [34]. The tools evaluated by this indicator were not specific for malaria, they included the HMIS registers, tally sheets and monthly summary forms, which are standard throughout the country.

Staff training on appropriate malaria case management scored lowest among the site readiness factors in both endemicity settings. We expected the scores to be high as the institutions (universities and colleges) training clinicians in Tanzania have standardised curricula that include the management of malaria. The low scores might be caused by how the assessment is conducted for this indicator. The MSDQI OPD checklist documents the number of available clinical staff, the number of staff who had received formal, or on-the-job training on malaria case management, including artesunate injectable prescription, and the mean of these scores is calculated. Injectable artesunate was introduced in Tanzania in 2013 and incorporated in the National Guidelines for Diagnosis and Treatment of Malaria in 2014 [35]. Facilities with a higher proportion of clinical staff who had graduated more than 8 years ago, before the introduction of artesunate, might score low in the first part of the checklist. Although on-the-job trainings on injection artesunate preparation and administration were provided when it was being introduced, a recent study shows that health care worker knowledge is still low [36].

### Clinical management

In both endemicity settings, health facilities scored the lowest in clinical management factors compared to the others assessed.

Facilities scored poorly in history taking and clinical examinations conducted for febrile patients in both endemicity settings. This might be explained by the human resource for health crisis in the country leading to a high workload [37]. The evaluation tool required the observer to assess fine details in history taking and physical examination that might have been skipped in a clinical setting with a high burden or turnover of patients. Patients might be referred quickly for laboratory confirmation without a thorough history and physical examination.

Health facilities scored higher in the testing rate for patients suspected to have malaria. However, there was no statistical difference in scores between the endemicity settings. This was not the case in Angola during a study which found there was a lower testing rate in low transmission settings [38].

In both endemicity settings, approximately 25% of the malaria test results (mRDT and microscopy) were not properly interpreted or the patient was diagnosed clinically to have malaria (i.e., without a test result). This proportion is lower compared to previous findings from the Service Provision Assessment for Malaria conducted in 2016 [39], but still reflects an unacceptably high number of facilities prescribing anti-malarial drugs for patients either not tested or having negative test results. A review of the HMIS data for the same period that this study was conducted showed that across the country approximately 3% of all malaria cases were diagnosed clinically without testing [40]. This practice is contrary to the national guidelines on malaria diagnosis and treatment, and the recommendations of WHO, which require treatment with anti-malarial drugs be limited to patients with parasitological confirmation of malaria infection [14, 17, 35].

Clinicians should improve on history taking skills and the performance of thorough physical examinations, refer for or conduct diagnostic tests for suspected malaria cases, and properly interpret and adhere to the test results before prescribing an appropriate antimalarial.

### Outcome factor

The level of patient satisfaction was high, comparable to that found in the Service Provision Assessment for Malaria, 2016, which reported satisfaction levels ranging from 60% to 85% [39]. It is important to note, however, that the Service Provision Assessment for Malaria did not calculate an overall satisfaction score. Patient satisfaction levels could have been influenced by differences in questionnaire length, content, and the potential for courtesy biases in the exit interviews conducted in both assessments. Patients were more satisfied with the services received in the mesoendemic regions compared to the hypoendemic regions. The checklist also gave scores when the patient could correctly explain the use of dispensed drugs at home, a situation that might favour the facilities in mesoendemic regions given they were more likely to prescribe medication as they attend more malaria cases.

### Strengths and limitations

Results from this study illustrated the quality of malaria case management services provided in outpatient departments across Tanzania mainland by utilising routine health facility assessment data obtained through the MSDQI package. This data was collected using a standardized approach that is sustainable. The approach aids in the collection, monitoring, and evaluation of malaria performance indicators at the facility level that provides timely, accurate information and data for decision making.

Findings from this study may serve as a baseline that could facilitate ongoing monitoring of case management performance at health facilities as MSDQI assessment data collection continues.

Countrywide analysis using the MSDQI OPD data before this study could not be performed. We merged MSDQI data from several sources including those that were collected using both paper-based and electronic tools that were initially stored in separate servers.

We assigned facilities to urban or rural locations; however, misclassification might have occurred in differentiating between facilities located in peri-urban settings.

Misclassification might have also occurred in differentiating endemicity settings by malaria prevalence estimates at the regional level. There is heterogeneity within regions. A better approach might be to perform the analysis at the district level and further differentiate the endemicity settings into more strata as previously defined using Tanzania HMIS and population survey data [41].

Cross-sectional health facility performance data that was collected between September 2017 and December 2018 was linked to estimates of malaria survey data conducted between October and December 2017. A better approach could have been to use data from real-time surveillance that would have enabled the detection of hot spots, especially in the hypoendemic regions.

The study analysed data from less than a quarter of all operating facilities in Tanzania mainland. The distribution of facilities across the country that were included was not uniform as some regions started the implementation of MSDQI supervisions earlier or had a higher rate of conducting supervisions than others. This might be influenced by the presence of implementing partners in the regions and the accessibility of the facilities by road for supervision during the dry and wet seasons. These might affect the generalisability of the results. As the coverage of the MSDQI assessments increases, more frequent analyses using data from all modules are warranted.

## Conclusion

Over 95% of all health facilities in our analysis sample scored *Good* or *Average* in the overall quality of case management. While there were differences observed in the quality of case management by endemicity, there is a need to target quality improvement, particularly in staff training and clinical management in the lower performing facilities, regardless of endemicity setting. Our methods and these findings could serve as a baseline for future analyses of Tanzania’s MSDQI data.

## Supporting information

S1 Checklist MSDQI OPD checklist. (PDF)

S1 Fig Distribution of overall quality scores of facilities by endemicity. (TIF)

S2 Fig Distribution of site readiness scores of facilities by endemicity. (TIF)

S3 Fig Distribution of clinical management scores of facilities by endemicity. (TIF)

S4 Fig Distribution of patient satisfaction scores of facilities by endemicity. (TIF)

S1 Table Health facility MSDQI OPD coverage by region. (DOCX)

S2 Table Distribution of all operating health facilities in Tanzania mainland by endemicity. (DOCX)

S1 Data (XLS)
