# Supplementary material for: The quality of malaria case management in different transmission settings in Tanzania mainland, 2017–2018
Source: PLOS Glob Public Health. 2023 Aug 21;3(8):e0002318. doi: 10.1371/journal.pgph.0002318 (PMC10441786; doi:10.1371/journal.pgph.0002318)
Supplement: S1 Checklist — (PDF) [file pgph.0002318.s001.pdf]

The United Republic of Tanzania

Ministry of Health, Community Development, Gender, Elderly and Children

National Malaria Control Program

Malaria Service and Data Quality Improvement Checklist:

Outpatient Department (OPD)

Section A. Health Facility Identifier

Council Name	____
Facility Name	____
Date of supervision visit	|dd|mm|yyyy|
Start time of supervision visit	|hh|mm| ☐ AM/ ☐ PM
Name of interviewer	____
Title of interviewer (Choose one)	**Regional:** ☐ Malaria and IMCI Focal Person ☐ Quality Improvement Focal Person ☐ Laboratory Technologist ☐ Pharmacist<br>**District:** ☐ Malaria and IMCI Focal Person ☐ Quality Improvement Focal Person ☐ Laboratory Technologist ☐ Pharmacist ☐ HMIS Focal Person ☐ RCH Coordinator<br>**Other:** ☐ Medical Officer in charge of the District Hospital ☐ Other, specify: ______________________________
Phone number of interviewer	+255______________________________________
Name of interviewee	____
Title of interviewee (Choose one)	☐ Head of Facility ☐ Medical Officer ☐ Laboratory Manager ☐ Head of OPD ☐ Pharmacist ☐ Laboratory Technologist ☐ Nurse<br>☐ Other, specify: ______________________________
Phone number of interviewee	+255______________________________________

Does this facility offer ANC services? ☐Yes ☐No

Has this facility conducted malaria microscopy at any point in the last three months? ☐Yes ☐No

If yes, is this facility offering this service today? ☐Yes ☐No

If no, why is this facility not conducting malaria microscopy today?

☐N/A – facility is conducting malaria microscopy today ☐Lack of supplies/equipment ☐Lack of power ☐ No lab staff who conduct malaria microscopy

Section B. List of core indicators and auxiliary indicators to be assessed

Core indicator	***Auxiliary indicator***
D. OPD readiness to properly manage malaria cases	*D1. OPD staffing*
	D2. Staff training
	D3. Malaria reference materials
	D4. Essential equipment
	D5. OPD Information system tools
E. Clinical management competence (observation)	*E1. Clinical History*
	E2. Physical Exam
	E3. Malaria Testing
	E4. Diagnosis
	E5. Treatment
	E6. Patient Counseling
F. Clients satisfaction	*F.1 Exit Interview*
G. OPD DQA	*G1. OPD-DQA Reporting Performance*
	G2.OPD-DQA Readiness
	G3. OPD-DQA consistency check
	G4. OPD sample register review

Section C. HMIS Indicator Review

C.1. HMIS Indicator Review

Instructions: Download and print out the previous quarter's HMIS data from the HMIS DHIS2 platform. Attach document or write in numbers below. Note: This information will not be entered into the MSDQI online platform.

Quarter: ☐ Jan-Mar, ☐ Apr-Jun, ☐ Jul-Sep, ☐ Oct-Dec Year ________________

Total number of OPD malaria cases	___________	% of malaria cases among total OPD attendance	_____________%
Total number of malaria patients dispensed ACTs	__________	% of confirmed malaria cases	_____________%
Malaria test rate	__________%		

Section C.2. Previous Supervision Visit Performance & Quality Improvement Plan

Instructions: Download and print out the previous supervision visit scores and quality improvement plan from the MSDQI DHIS2 platform. Attach document or write in quality improvement plan below.

Date of previous visit: |dd|mm|yyyy|

Checklist Section	Score	Describe the impediment	What immediate action was done?	What was the action plan?	Who was the person responsible?	What was the time frame?	Was this gap addressed?	If addressed, explain action taken. If partially or not addressed, describe the main barriers:
☐OPD Site Readiness<br>☐OPD Observations<br>☐Patient Satisfaction<br>☐OPD-DQA	_____%						☐Completely addressed<br>☐Partially addressed<br>☐Not addressed	
☐OPD Site Readiness<br>☐OPD Observations<br>☐Patient Satisfaction<br>☐OPD-DQA	_____%						☐Completely addressed<br>☐Partially addressed<br>☐Not addressed	
☐OPD Site Readiness<br>☐OPD Observations<br>☐Patient Satisfaction<br>☐OPD-DQA	_____%						☐Completely addressed<br>☐Partially addressed<br>☐Not addressed	

Section D. OPD Site Readiness

D.1 Staffing Levels

Instructions: Determine the number of staff members present on the day of the visit for the facility depending on its level. Only fill out the appropriate section based on the level of the facility and leave the remaining sections blank. The score will be calculated based on the facility level only.

Is this facility a dispensary?<br>**If yes:**	☐Yes	☐No
Is there at least one (1) Clinical Officer/Assistant available in this OPD?	☐Yes [3]	☐No [0]
Is there at least one (1) Nurse available in this OPD?	☐Yes [3]	☐No [0]
Is this facility a health center or hospital?<br>**If yes:**	☐Yes	☐No
Is there at least one (1) Medical Doctor available in this OPD?	☐Yes [1]	☐No [0]
Is there at least one (1) Assistant Medical Officer available in this OPD?	☐Yes [1]	☐No [0]
Are there at least two (2) Clinical Officers available in this OPD?	☐Yes [1]	☐No [0]
Is there at least one (1) Assistant Nursing Officer available in this OPD?	☐Yes [1]	☐No [0]
Is there at least one (1) Nurse available in this OPD?	☐Yes [1]	☐No [0]
Is there at least one (1) Medical Attendant available in this OPD?	☐Yes [1]	☐No [0]
**SUB-SCORE: D.1 Staffing Levels**	**[__]/6 =**	**_____%**

D.2 Staff Training

How many total clinical staff are at this OPD?	A.____________
How many clinical staff received formal (e.g seminar) malaria case management training including artesunate injectable prescription?	B.____________<br>**C. B/A=_______%**
How many clinical staff received on-job (e.g mentorship) malaria case management training including artesunate injectable prescription? *(verify record of the training)*	D.____________<br>**E. D/A=_______%**
**SUB-SCORE: D.2 Staff Training**	**[C+E]/2= _______%**

D.3 Malaria Reference Materials *(full score = available and accessible, partial score = available but not accessible, 0 = not available)*

Are the following available in the OPD? Please verify.			
2014 Malaria Diagnosis and Treatment Guideline	☐Yes [2]	☐Partial [1]	☐No [0]
2014-15 Training manual	☐Yes [2]	☐Partial [1]	☐No [0]
2016 IMCI Chart booklet	☐Yes [2]	☐Partial [1]	☐No [0]
Fever case management algorithm poster	☐Yes [2]	☐Partial [1]	☐No [0]
Artesunate injection job aid/SOP	☐Yes [2]	☐Partial [1]	☐No [0]
**SUB-SCORE: D.3 Malaria Reference Materials**	**[___]/10 =**		**_____%**

D.4 Essential Equipment *(full score = available and functional, partial score = available but not functional, 0 = not available)*

Are the following available and functioning in this OPD?			
Thermometer	☐Yes [2]	☐Partial [1]	☐No [0]
Timing device (e.g. ARI timer)	☐Yes [2]	☐Partial [1]	☐No [0]
Stethoscope	☐Yes [2]	☐Partial [1]	☐No [0]
BP machine	☐Yes [2]	☐Partial [1]	☐No [0]
Weighing scale	☐Yes [2]	☐Partial [1]	☐No [0]
**SUB-SCORE: D.4 Essential Equipment**		**[___]/10 =**	**_____%**

D.5 OPD Information System Tools

Check the following information system tools:

**Information System**	**Available**		**Standard Format**	
**MTUHA Register #5**	☐Yes [2]	☐No [0]	☐Yes [2]	☐No [0]
**Tally sheets**	☐Yes [2]	☐No [0]	☐Yes [2]	☐No [0]
**Monthly summary**	☐Yes [2]	☐No [0]	☐Yes [2]	☐No [0]
**SUB-SCORE: D.5 OPD Information System Tools**			**[___]/12 =**	**_____%**

Section D. OPD Site Readiness: Score

Instructions: Calculate Total Section Score by averaging section Scores				
D.1 Staffing Levels	D.2 Staff Training	D.3 Malaria Reference Materials	D.4 Essential Equipment	D.5 OPD Information System Tools
_____%	_____%	_____%	_____%	_____%

SCORE: Section D. OPD Site Readiness	[______]/5 = _____%

Section E. OPD Observations:

E.1: Patients <5 years of age

Instructions: Observe the health service provider while attending under five patients with fever. If a facility has more than one health service provider doing consultations, observations should be made to different health service providers. If there is only one health service provider doing consultations then all observations should be done on the available health service provider. Do not interrupt unless the patient is severely ill or if the practice of the clinician will put the patient in danger. If a patient is severely ill then the supervisor should assist in giving treatment while doing mentorship to the available clinicians and other health service providers. (N = number of observations)

HEALTH PROVIDER INFORMATION	Observation 1		Observation 2	
What is the cadre of the observed health provider?	☐Clinician<br>☐Nurse Officer/Assistant Nurse Officer/Enrolled Nurse<br>☐Medical Attendant<br>☐Other		☐Clinician<br>☐ Nurse Officer/Assistant Nurse Officer/Enrolled Nurse<br>☐Medical Attendant<br>☐Other	
Has this health provider received training in malaria case management and/or IMCI?	☐Yes	☐No	☐Yes	☐No
**If yes,** type of training received	☐Formal class	☐On the job	☐Formal class	☐On the job
**If yes,** what year did the training occur?	________________		________________	
**E1.1 CLINICAL HISTORY**	**Observation 1**		**Observation 2**	
Did the health provider ask/check the following?				
Age of patient	☐Yes [1]	☐No [0]	☐Yes [1]	☐No [0]
Fever	☐Yes [0.5]	☐No [0]	☐Yes [0.5]	☐No [0]
Duration of fever (Answer no if clinician did not ask about fever)	☐Yes [0.5]	☐No [0]	☐Yes [0.5]	☐No [0]
Diarrhea	☐Yes [0.5]	☐No [0]	☐Yes [0.5]	☐No [0]

	Observation 1		Observation 2	
Asked if diarrhea was bloody (N/A if patient does not have diarrhea) (No if clinician did not ask about diarrhea)	☐Yes [0.5]	☐No [0]	☐Yes [0.5]	☐No [0]
	☐N/A [0.5]		☐N/A [0.5]	
Cough	☐Yes [0.5]	☐No [0]	☐Yes [0.5]	☐No [0]
Duration of cough (N/A if patient does not have cough) (No if clinician did not ask about cough)	☐Yes [0.5]	☐No [0]	☐Yes [0.5]	☐No [0]
	☐N/A [0.5]		☐N/A [0.5]	
Ear problems	☐Yes [1]	☐No [0]	☐Yes [1]	☐No [0]
Vomiting everything	☐Yes [1]	☐No [0]	☐Yes [1]	☐No [0]
Not able to drink or breastfeed	☐Yes [1]	☐No [0]	☐Yes [1]	☐No [0]
History of convulsions in this illness or convulsing now	☐Yes [1]	☐No [0]	☐Yes [1]	☐No [0]
Altered consciousness or coma	☐Yes [1]	☐No [0]	☐Yes [1]	☐No [0]
Treatment given prior to arrival at a facility	☐Yes [1]	☐No [0]	☐Yes [1]	☐No [0]
**SUB-SCORE**	**A. [______]**		**B. [______]**	
**SUB-SCORE: E.1.1 Clinical History**	**[A+B] = _________/10N=______%**			
**E1.2 PHYSICAL EXAMINATION**	**Observation 1**		**Observation 2**	
Did the health provider check for the following?				
Weight of the patient	☐Yes [2]	☐No [0]	☐Yes [2]	☐No [0]
Evidence of anaemia (Palmar/conjunctiva/tongue pallor)?	☐Yes [2]	☐No [0]	☐Yes [2]	☐No [0]
Temperature taken	☐Yes [2]	☐No [0]	☐Yes [2]	☐No [0]
ENT examination and/or respiratory rate	☐Yes [1]	☐No [0]	☐Yes [1]	☐No [0]
Evidence of convulsion	☐Yes [1]	☐No [0]	☐Yes [1]	☐No [0]
Evidence of altered consciousness or coma. Health service provider measures patient's alertness, voice, pain and unresponsiveness (AVPU)	☐Yes [1]	☐No [0]	☐Yes [1]	☐No [0]
Neck exam (stiffness)	☐Yes [1]	☐No [0]	☐Yes [1]	☐No [0]
**SUB-SCORE**	**C. [______]**		**D. [______]**	
**SUB-SCORE: E.1.2 Physical Exam**	**[C+D] = _________/10N=______%**			
**E1.3 MALARIA TESTING**	**Observation 1**		**Observation 2**	
Does the health service provider order/conduct a malaria test?	☐Yes [5]	☐No [0]	☐Yes [5]	☐No [0]
**If yes,** does the health service provider wait for the test results before the final diagnosis/prescription?	☐Yes [5]	☐No [0]	☐Yes [5]	☐No [0]

**If no,** was the test not done because of one of the following reasons?<br>**[IF THE PATIENT WAS TESTED, LEAVE BLANK]**	☐ No RDT/microscopy available [5]<br>☐ Patient had signs of severe febrile illness [5]<br>☐ Other reason [0]	☐ No RDT/microscopy available [5]<br>☐ Patient had signs of severe febrile illness [5]<br>☐ Other reason [0]
**SUB-SCORE**	**E. [______]**	**F. [______]**
**SUB-SCORE: E1.3 Malaria Testing**	**[E+F] = _________/20N=______%**	
**<u>E1.4 DIAGNOSIS</u>**	**<u>Observation 1</u>**	**<u>Observation 2</u>**
Malaria test results	☐ Positive ☐ Negative<br>☐ Test result not available<br>☐ Not tested	☐ Positive ☐ Negative<br>☐ Test result not available<br>☐ Not tested
Was there a clinical or confirmed malaria diagnosis?	☐ Clinical malaria<br>☐ Confirmed malaria (mRDT or BS +ve)<br>☐ Other febrile illness (no malaria diagnosis)	☐ Clinical malaria<br>☐ Confirmed malaria (mRDT or BS +ve)<br>☐ Other febrile illness (no malaria diagnosis)
Did the health service provider make correct diagnosis according to National Guidelines for Malaria Diagnosis and Treatment?<br>- If positive malaria test (Pos), diagnosis to be *malaria confirmed* = <u>*yes*</u> (other febrile illnesses might also be <u>yes</u>)<br>- If negative, malaria test (Neg), diagnosis to be *other febrile illnesses* = <u>*yes*</u><br>- If malaria test was not done (NT) or result not available (N/A), all diagnosis are acceptable except *malaria confirmed* = <u>*yes*</u>	☐ Yes [10] ☐ No [0]	☐ Yes [10] ☐ No [0]
**SUB-SCORE**	**G. [______]**	**H. [______]**
**SUB-SCORE: E1.4 Diagnosis**	**[G+H] = _________/10N=______%**	
**<u>E1.5 TREATMENT</u>**	**<u>Observation 1</u>**	**<u>Observation 2</u>**
Was the patient diagnosed with malaria?	☐ Yes ☐ No	☐ Yes ☐ No
**If yes,** was an ACT given?	☐ Yes [10] ☐ No [0]	☐ Yes [10] ☐ No [0]
**If yes,** was ACT dosage prescribed according to body weight or age if applicable?	☐ Yes [5] ☐ No [0]	☐ Yes [5] ☐ No [0]

**If yes,** was ACT regimen prescribed according to recommended frequency (e.g. for ALu twice per day for three days)?	☐ Yes [5]	☐ No [0]	☐ Yes [5]	☐ No [0]
Was the patient diagnosed as **NOT** having malaria?	☐ Yes	☐ No	☐ Yes	☐ No
**If yes,** was an ACT **not** given?	☐ Yes [10]	☐ No [0]	☐ Yes [10]	☐ No [0]
**If yes,** was the medication dosage for non-malaria diagnosis prescribed according to body weight or age if applicable?	☐ Yes [5]	☐ No [0]	☐ Yes [5]	☐ No [0]
**If yes,** was the medication regimen for non-malaria diagnosis prescribed according to the recommended frequency (e.g. for Amoxicillin 500 mg TDS for 5 days)?	☐ Yes [5]	☐ No [0]	☐ Yes [5]	☐ No [0]
**SUB-SCORE**	**I. [______]**		**J. [______]**	
**SUB-SCORE: E1.5 Treatment**	**[I+J] = _________/40N=______%**			

**E1.6 PATIENT COUNSELING**	**Observation 1**		**Observation 2**	
Does the provider discuss the following with the patient:				
How to give/take medicines at home?	☐Yes [2]	☐No [0]	☐Yes [2]	☐No [0]
When to return?	☐Yes [2]	☐No [0]	☐Yes [2]	☐No [0]
Use of LLIN?	☐Yes [2]	☐No [0]	☐Yes [2]	☐No [0]
Checked to confirm understanding of client?	☐Yes [2]	☐No [0]	☐Yes [2]	☐No [0]
Asked if client has any questions?	☐Yes [2]	☐No [0]	☐Yes [2]	☐No [0]
**SUB-SCORE**	**K. [______]**		**L. [______]**	
**SUB-SCORE: E1.6 Patient Counselling**	**[K+L] = _________/10N=______%**			

Reason if unable to complete observation:<br>1. N/A – Observation is complete<br>2. No febrile patient available<br>3. No clinician available<br>4. Patient referred out<br>5. Stopped consultation due to potential patient harm<br>6. Not enough time during facility visit<br>7. Other (explain)	☐<br>Other:_____________	☐<br>Other:_____________

Section E. OPD Observations:

E.2: Patients >=5 years of age

Instructions: Observe the service health provider while attending patients over five with fever. If a facility has more than one consultation room, observations should be made to different health service providers, if there is only one consultation room then all observations should be done in that available health care provider. Do not interrupt unless the patient is severely ill or if the practice of the clinician will put the client in danger. If a patient is severely ill then the supervisor should assist in giving treatment while doing mentorship to the available clinicians and other health service providers. (N = number of observations)

**HEALTH PROVIDER INFORMATION**	**Observation 3**	**Observation 4**
What is the cadre of the observed health provider?	☐Clinician<br>☐ Nurse Officer/Assistant Nurse Officer/Enrolled Nurse<br>☐Medical Attendant<br>☐Other	☐Clinician<br>☐ Nurse Officer/Assistant Nurse Officer/Enrolled Nurse<br>☐Medical Attendant<br>☐Other
Has this health provider received training in malaria case management and/or IMCI?	☐Yes ☐No	☐Yes ☐No
**If yes,** type of training received	☐Formal class ☐On the job	☐Formal class ☐On the job
**If yes,** what year did the training occur?	___________________	___________________
**E2.1 CLINICAL HISTORY**	**Observation 3**	**Observation 4**
Did the health provider ask/check the following?		
Whether the patient is pregnant? (if female 15-49)	☐Yes [1] ☐No [0] ☐N/A [1]	☐Yes [1] ☐No [0] ☐N/A [1]
Fever	☐Yes [0.5] ☐No [0]	☐Yes [0.5] ☐No [0]
Duration of fever<br>(Answer no if clinician did not ask	☐Yes [0.5] ☐No [0]	☐Yes [0.5] ☐No [0]

about fever)		
Cough	☐Yes [0.5] ☐No [0]	☐Yes [0.5] ☐No [0]
Duration of cough (N/A if patient does not have cough)	☐Yes [0.5] ☐No [0] ☐N/A [0.5]	☐Yes [0.5] ☐No [0] ☐N/A [0.5]
Headache; joint pain/body ache	☐Yes [1] ☐No [0]	☐Yes [1] ☐No [0]
Dizziness/malaise	☐Yes [1] ☐No [0]	☐Yes [1] ☐No [0]
History of convulsion	☐Yes [1] ☐No [0]	☐Yes [1] ☐No [0]
Behavioral change	☐Yes [1] ☐No [0]	☐Yes [1] ☐No [0]
Severe abdominal pain	☐Yes [1] ☐No [0]	☐Yes [1] ☐No [0]
Treatment given prior to arrival at the facility	☐Yes [1] ☐No [0]	☐Yes [1] ☐No [0]
Drug allergy	☐Yes [1] ☐No [0]	☐Yes [1] ☐No [0]
**SUB-SCORE**	**A. [______]**	**B. [______]**
**SUB-SCORE: E2.1 Clinical History**	**[A+B] = __________/10N=______%**	
**E2.2 PHYSICAL EXAMINATION**	**Observation 3**	**Observation 4**
Did the health provider check for the following?		
Weight and temperature recorded/taken	☐Yes [2] ☐No [0]	☐Yes [2] ☐No [0]
Evidence of anaemia (Palmar/conjunctiva/tongue pallor and/or jaundice)?	☐Yes [2] ☐No [0]	☐Yes [2] ☐No [0]
ENT examination	☐Yes [2] ☐No [0]	☐Yes [2] ☐No [0]
All other system examinations (chest, abdomen, limbs)	☐Yes [2] ☐No [0]	☐Yes [2] ☐No [0]
At least one sign of severe disease: respiratory distress, altered consciousness or coma (eye opened, verbal and motor response), neck exam (stiffness)	☐Yes [2] ☐No [0]	☐Yes [2] ☐No [0]
**SUB-SCORE**	**C. [______]**	**D. [______]**
**SUB-SCORE: E.2.2 Physical Exam**	**[C+D] = __________/10N=______%**	
**MALARIA TESTING**	**Observation 3**	**Observation 4**
Does the health service provider order/conduct a malaria test?	☐Yes [5] ☐No [0]	☐Yes [5] ☐No [0]
**If yes,** does the health service provider wait for the test results before the final diagnosis/prescription?	☐Yes [5] ☐No [0]	☐Yes [5] ☐No [0]

**If no,** was the test not done because of one of the following reasons?<br>**[IF THE PATIENT WAS TESTED, LEAVE BLANK]**	☐ No RDT or microscopy available [5]<br>☐ Patient had signs of severe febrile illness [5]<br>☐ Other reason [0]	☐ No RDT or microscopy available [5]<br>☐ Patient had signs of severe febrile illness [5]<br>☐ Other reason [0]
**SUB-SCORE**	**E. [______]**	**F. [______]**
**SUB-SCORE: E.2.3 Malaria Testing**	**[E+F] = _________/20N=______%**	
**E2.3 DIAGNOSIS**	**Observation 3**	**Observation 4**
Malaria test results	☐ Positive ☐ Negative<br>☐ Test result not available<br>☐ Not tested	☐ Positive ☐ Negative<br>☐ Test result not available<br>☐ Not tested
Was there a clinical or confirmed malaria diagnosis?	☐ Clinical malaria<br>☐ Confirmed malaria (mRDT or BS +ve)<br>☐ Other febrile illness (no malaria diagnosis)	☐ Clinical malaria<br>☐ Confirmed malaria (mRDT or BS +ve)<br>☐ Other febrile illness (no malaria diagnosis)
Did the health service provider make correct diagnosis according to National Guidelines for Malaria Diagnosis and Treatment?<br>- If positive malaria test (Pos), diagnosis to be *malaria confirmed = yes* (other febrile illnesses might also be yes)<br>- If negative, malaria test (Neg), diagnosis to be *other febrile illnesses = yes*<br>- If malaria test was not done (NT) or result not available (N/A), all diagnosis are acceptable except *malaria confirmed = yes*	☐ Yes [10] ☐ No [0]	☐ Yes [10] ☐ No [0]
**SUB-SCORE**	**G. [______]**	**H. [______]**
**SUB-SCORE: E.2.4 Diagnosis**	**[G+H] = _________/10N=______%**	
**E2.5 TREATMENT**	**Observation 3**	**Observation 4**
Was the patient diagnosed with malaria?	☐ Yes ☐ No	☐ Yes ☐ No
**If yes,** was an ACT given?	☐ Yes [10] ☐ No [0]	☐ Yes [10] ☐ No [0]

**If yes,** was ACT dosage prescribed according to body weight or age if applicable?	☐ Yes [5] ☐ No [0]	☐ Yes [5] ☐ No [0]
**If yes,** was ACT regimen prescribed according to recommended frequency (e.g. for ALu twice per day for three days)?	☐ Yes [5] ☐ No [0]	☐ Yes [5] ☐ No [0]
Was the patient diagnosed as **NOT** having malaria?	☐ Yes ☐ No	☐ Yes ☐ No
**If yes,** was an ACT not given?	☐ Yes [10] ☐ No [0]	☐ Yes [10] ☐ No [0]
**If yes,** was the medicament dosage for no malaria diagnosis prescribed according to body weight or age if applicable?	☐ Yes [5] ☐ No [0]	☐ Yes [5] ☐ No [0]
**If yes,** was the medicament regimen for no malaria diagnosis prescribed according to the recommended frequency (e.g. for Amoxicillin 500 mg TDS for 5 days)?	☐ Yes [5] ☐ No [0]	☐ Yes [5] ☐ No [0]
**SUB-SCORE**	**I. [______]**	**J. [______]**
**SUB-SCORE: E.2.5 Treatment**	**[I+J] = _________/40N=______%**	
**E2.6 PATIENT COUNSELING**	**Observation 3**	**Observation 4**
Does the provider discuss the following with the patient:		
How to give/take medicines at home?	☐Yes [2] ☐No [0]	☐Yes [2] ☐No [0]
When to return?	☐Yes [2] ☐No [0]	☐Yes [2] ☐No [0]
Use of LLIN?	☐Yes [2] ☐No [0]	☐Yes [2] ☐No [0]
Checked to confirm understanding of client?	☐Yes [2] ☐No [0]	☐Yes [2] ☐No [0]
Asked if client has any questions?	☐Yes [2] ☐No [0]	☐Yes [2] ☐No [0]
**SUB-SCORE**	**K. [______]**	**L. [______]**
**SUB-SCORE: E.2.6 Patient Counselling**	**[K+L] = _________/10N=______%**	
Reason if unable to complete RDT observation:<br>1. N/A – Observation is complete<br>2. No febrile patient available<br>3. No health service provider available<br>4. Patient referred out<br>5. Stopped consultation due to potential patient harm<br>6. Not enough time during facility visit<br>7. Other (explain)	☐<br>Other:_____________	☐<br>Other:_____________

Section E. OPD Observations: Score

Instructions: Calculate Total Section Score by averaging section Scores			
	Patient Under 5	Patients Over 5	Total
E1. Clinical History	E.1.1: _______%	E.2.1: _______%	[E.1.1+E.2.1]/N=________%
E2. Physical Exam	E.1.2: _______%	E.2.2: _______%	[E.1.2+E.2.2]/N=________%
E3. Malaria Testing	E.1.3: _______%	E.2.3: _______%	[E.1.3+E.2.3]/N=________%
E4. Diagnosis	E.1.4: _______%	E.2.4: _______%	[E.1.4+E.2.4]/N=________%
E5. Treatment	E.1.5: _______%	E.2.5: _______%	[E.1.5+E.2.5]/N=________%
E6. Patient Counseling	E.1.6: _______%	E.2.6: _______%	[E.1.6+E.2.6]/N=________%
SCORE: Section E. OPD Observations		[E1-E6] = _________/6= _____%	

Section F. Exit Interview

Instructions: Ask 2 different patients/caretakers that are about to leave the facility. These patients should have been tested for malaria, diagnosed to have malaria, or prescribed antimalarial (N=number of interviews)		
	Interview 1	Interview 2
Did you get all the medicines prescribed for your illness at this facility?	☐Yes [2]<br>☐Partially [1]<br>☐No [0]	☐Yes [2]<br>☐Partially [1]<br>☐No [0]
If the response to above question is Yes or partial then ask the patient to explain how to use the medicines?<br>If the response to above question is no, mark no in the following question		
Supervisor, could the client/caretaker explain use of dispensed drugs at home correctly?	☐Yes [2]<br>☐Partially [1]<br>☐No [0]	☐Yes [2]<br>☐Partially [1]<br>☐No [0]
Ask patient/care taker: “Can you explain to me when you should return to the health facility?”		
Supervisor, could the client/caretaker explain when to return to health correctly?	☐Yes [2]<br>☐Partially [1]<br>☐No [0]	☐Yes [2]<br>☐Partially [1]<br>☐No [0]
Ask patient/care taker: “Can you explain to me when you should return to the health facility?”		
Supervisor, could the client/caretaker explain when to return to health correctly?	☐Yes [2]<br>☐Partially [1]<br>☐No [0]	☐Yes [2]<br>☐Partially [1]<br>☐No [0]
Are you satisfied with the time you spent to receive services today?	☐Yes [2]<br>☐Partially [1]<br>☐No [0]	☐Yes [2]<br>☐Partially [1]<br>☐No [0]
Are you satisfied with the services provided by the health facility staff?	☐Yes [2]<br>☐Partially [1]<br>☐No [0]	☐Yes [2]<br>☐Partially [1]<br>☐No [0]
TOTAL SCORE:<br>Section F Client Satisfaction	A. [_____]<br>[A+B]/12N=______%	B. [_____]

Section G. OPD-DQA Module

Instructions: ONLY Complete Section G if there is no separate DQA supervision during this visit. Note: For electronic data entry enter this section into the "OPD-DQA" module.

Section G.1. OPD-DQA Reporting Performance

**Instructions: Review the forms available at the facility and the dates of submission to verify the following items.**	
Did this facility submit the following malaria reports to DHIS2 for the most recent reporting months?	
OPD report form	☐Yes [2]<br>☐No [0]
Dispensing report form	☐Yes [2]<br>☐No [0]
Malaria testing report form	☐Yes [2]<br>☐No [0]
IDSR report	☐Yes [2]<br>☐No [0]
Does this facility submit reports to CHMT?	☐Yes<br>☐No
**If yes,** was the facility report submitted to CHMT on or before 7th of the subsequent month?	☐Yes [2]<br>☐No [0]
Does this facility submit reports directly to DHIS2?	☐Yes<br>☐No
**If yes,** was report entered into DHIS2 on or before 15th of the subsequent month?	☐Yes [2]<br>☐No [0]
**SCORE: Section G.1 OPD-DQA Reporting Performance**	**[_____]/10=______%**

Section G.2. OPD-DQA Readiness

**Instructions: Interviews the person or person(s) involved in data entry and management at the facility to complete this section.**	
The responsibility of recording the delivery of service on monthly summary forms for OPD, Dispensing and Laboratory is clearly assigned to relevant staff	☐Yes [2]<br>☐No [0]
There are designated staff at the facility responsible for reviewing aggregated number prior to submission to the council level	☐Yes [2]<br>☐No [0]
**All** staff involved in recording on monthly summary forms have received training on the data management and tools	☐Yes [2]<br>☐No [0]
The facility has written guidelines (MUONGOZO #1) to the service delivery point on reporting requirements and deadlines	☐Yes [2]<br>☐No [0]
Malaria data used at facility meetings for planning and decision making<br>***If yes, discuss with staff how data is used to verify.***	☐Yes [2]<br>☐No [0]
**SCORE: Section G.2 OPD-DQA Data Readiness**	**[_____]/10=______%**

Section G.3. OPD-DQA Consistency Check

Instructions: Choose the last month from the most recent quarter for which DHIS2 data has been submitted. Review the Register, Tally and Summary forms in the OPD, Dispensing and Laboratory Register and write the values in each of the blank squares. Compare the values in the various forms as directed in the table below. Give 1 point for each of the matching numbers.

#		OPD			DISPENSING			LABORATORY			DHIS2				Check 1 (Register – Tally)	Check 2 (Tally-Summary)/	Check 3 (Summary-DHIS2)	Check 4 (Lab / Dispensing Summary - OPD Summary)	Total Score
	**MONTH:** _____/____ [MMM/YYYY]	Register	Tally	Summary	Register	Tally	Summary	Register	Tally	Summary	R&R	OPD	Lab.	Disp.					
	**DATA ELEMENTS**	A	B	C	D	E	F	G	H	I	J	K	L	M					
1	OPD attendance														**A=B?** ☐Y [1] ☐N [0]	**B=C?** ☐Y [1] ☐N [0]	**C=K?** ☐Y [1] ☐N [0]		_____
2	Clinical Malaria														**A=B?** ☐Y [1] ☐N [0]	**B=C?** ☐Y [1] ☐N [0]	**C=K?** ☐Y [1] ☐N [0]		_____
3	Malaria BS +ve ☐N/A – No BS at facility														**A=B?** ☐Y [1] ☐N [0] ☐N/A [1]	**B=C?** ☐Y [1] ☐N [0] ☐N/A [1]	**C=K?** ☐Y [1] ☐N [0] ☐N/A [1]	**I=C?** ☐Y [1] ☐N [0] ☐N/A [1]	_____
															**G=H?** ☐Y [1] ☐N [0] ☐N/A [1]	**I=H?** ☐Y [1] ☐N [0] ☐N/A [1]			_____
4	Malaria mRDT +ve														**A=B?** ☐Y [1] ☐N [0]	**B=C?** ☐Y [1] ☐N [0]	**C=K?** ☐Y [1] ☐N [0]	**I=C?** ☐Y [1] ☐N [0]	_____
															**G=H?** ☐Y [1] ☐N [0]	**I=H?** ☐Y [1] ☐N [0]			_____
5	Patients tested with mRDTs														**G=H?** ☐Y [1] ☐N [0]	**I=H?** ☐Y [1] ☐N [0]	**I=L?** ☐Y [1] ☐N [0]	**A=I?** ☐Y [1] ☐N [0]	_____
6	Patients tested with BS ☐N/A – No BS at facility														**G=H?** ☐Y [1] ☐N [0] ☐N/A [1]	**I=H?** ☐Y [1] ☐N [0] ☐N/A [1]	**I=L?** ☐Y [1] ☐N [0] ☐N/A [1]	**A=I?** ☐Y [1] ☐N [0] ☐N/A [1]	_____
7	Patients prescribed/ dispensed ACT														**D=E?** ☐Y [1] ☐N [0]	**E=F?** ☐Y [1] ☐N [0]	**F=M?** ☐Y [1] ☐N [0]	**A=F?** ☐Y [1] ☐N [0]	_____
	**SCORE: Section G3. OPD-DQA Consistency Check [______]/30 = ___________%**																		

Section G.4. OPD Sample Register Review

Instructions: Select the first ten patients from the start of the past month OPD register who have the following criteria: a) were prescribed antimalarial and/or antibiotic, b) tested for malaria, or c) with malaria diagnosis. Then list them (name/identification/age/sex) on a separate paper and track their information to see if they are recorded in the testing register and dispending register – a template has been included at the end of this document for this purpose. "Match OPD" columns are marked if the test results in the malaria test register (MAL: Results) is found AND matches the OPD register (OPD: Result) and if the ACT prescription in the Dispensing Register (DIST: ACT) is found AND matches the OPD register (OPD: ACT).

Once the information has been entered, destroy the paper with names by ripping up and discarding it for confidentiality.

AMX = Anti-malarial treatment, ABX = Antibiotic treatment, ASA = Acetyl Salicilic Acid, PCM = Paracetamol, MAL = Malaria, ACT = Artemisinin Combination Therapy

#	OPD Register										Malaria Test Register	Dispensing Register	
	OPD: Age	OPD: Test Order	OPD: Res.	OPD: Diagnosis	OPD: Results Match Diag	OPD: AMX	OPD: AMX Match Diag	OPD: PCM/ ASA	OPD: ABX	OPD: Other	MAL: Results	DIS: ACT	DIS: ACT Match MAL: Res
1	☐U5<br>☐ 5+<br>☐ NR	☐Y [1]<br>☐N [0]	☐Pos<br>☐Neg<br>☐NR	☐Mal<br>☐Not Mal<br>☐ NR	☐Y [1]<br>☐N [0]<br>☐NR [0]	☐ACT<br>☐Artes. Inj<br>☐Other<br>☐ NR	☐Y [1]<br>☐N [0]	☐Y<br>☐N	☐Y<br>☐N	☐Y<br>☐N	☐Pos<br>☐Neg<br>☐NF	☐Y<br>☐N<br>☐NF	☐Y [1]<br>☐N [0]
2	☐U5<br>☐ 5+<br>☐ NR	☐Y [1]<br>☐N [0]	☐Pos<br>☐Neg<br>☐NR	☐Mal<br>☐Not Mal<br>☐ NR	☐Y [1]<br>☐N [0]<br>☐NR [0]	☐ACT<br>☐Artes. Inj<br>☐Other<br>☐ NR	☐Y [1]<br>☐N [0]	☐Y<br>☐N	☐Y<br>☐N	☐Y<br>☐N	☐Pos<br>☐Neg<br>☐NF	☐Y<br>☐N<br>☐NF	☐Y [1]<br>☐N [0]
3	☐U5<br>☐ 5+<br>☐ NR	☐Y [1]<br>☐N [0]	☐Pos<br>☐Neg<br>☐NR	☐Mal<br>☐Not Mal<br>☐ NR	☐Y [1]<br>☐N [0]<br>☐NR [0]	☐ACT<br>☐Artes. Inj<br>☐Other<br>☐ NR	☐Y [1]<br>☐N [0]	☐Y<br>☐N	☐Y<br>☐N	☐Y<br>☐N	☐Pos<br>☐Neg<br>☐NF	☐Y<br>☐N<br>☐NF	☐Y [1]<br>☐N [0]
4	☐U5<br>☐ 5+<br>☐ NR	☐Y [1]<br>☐N [0]	☐Pos<br>☐Neg<br>☐NR	☐Mal<br>☐Not Mal<br>☐ NR	☐Y [1]<br>☐N [0]<br>☐NR [0]	☐ACT<br>☐Artes. Inj<br>☐Other<br>☐ NR	☐Y [1]<br>☐N [0]	☐Y<br>☐N	☐Y<br>☐N	☐Y<br>☐N	☐Pos<br>☐Neg<br>☐NF	☐Y<br>☐N<br>☐NF	☐Y [1]<br>☐N [0]
5	☐U5<br>☐ 5+<br>☐ NR	☐Y [1]<br>☐N [0]	☐Pos<br>☐Neg<br>☐NR	☐Mal<br>☐Not Mal<br>☐ NR	☐Y [1]<br>☐N [0]<br>☐NR [0]	☐ACT<br>☐Artes. Inj<br>☐Other<br>☐ NR	☐Y [1]<br>☐N [0]	☐Y<br>☐N	☐Y<br>☐N	☐Y<br>☐N	☐Pos<br>☐Neg<br>☐NF	☐Y<br>☐N<br>☐NF	☐Y [1]<br>☐N [0]

#		OPD Register									Malaria Test Register	Dispensing Register	
	OPD: Age	OPD: Test Order	OPD: Res.	OPD: Diagnosis	OPD: Results Match Diag	OPD: AMX	OPD: AMX Match Diag	OPD: PCM/ ASA	OPD: ABX	OPD: Other	MAL: Results	DIS: ACT	DIS: ACT Match MAL: Res
6	☐U5<br>☐ 5+<br>☐ NR	☐Y [1]<br>☐N [0]	☐Pos<br>☐Neg<br>☐NR	☐Mal<br>☐Not Mal<br>☐ NR	☐Y [1]<br>☐N [0]<br>☐NR [0]	☐ACT<br>☐Artes. Inj<br>☐Other<br>☐ NR	☐Y [1]<br>☐N [0]	☐Y<br>☐N	☐Y<br>☐N	☐Y<br>☐N	☐Pos<br>☐Neg<br>☐NF	☐Y<br>☐N<br>☐NF	☐Y [1]<br>☐N [0]
7	☐U5<br>☐ 5+<br>☐ NR	☐Y [1]<br>☐N [0]	☐Pos<br>☐Neg<br>☐NR	☐Mal<br>☐Not Mal<br>☐ NR	☐Y [1]<br>☐N [0]<br>☐NR [0]	☐ACT<br>☐Artes. Inj<br>☐Other<br>☐ NR	☐Y [1]<br>☐N [0]	☐Y<br>☐N	☐Y<br>☐N	☐Y<br>☐N	☐Pos<br>☐Neg<br>☐NF	☐Y<br>☐N<br>☐NF	☐Y [1]<br>☐N [0]
8	☐U5<br>☐ 5+<br>☐ NR	☐Y [1]<br>☐N [0]	☐Pos<br>☐Neg<br>☐NR	☐Mal<br>☐Not Mal<br>☐ NR	☐Y [1]<br>☐N [0]<br>☐NR [0]	☐ACT<br>☐Artes. Inj<br>☐Other<br>☐ NR	☐Y [1]<br>☐N [0]	☐Y<br>☐N	☐Y<br>☐N	☐Y<br>☐N	☐Pos<br>☐Neg<br>☐NF	☐Y<br>☐N<br>☐NF	☐Y [1]<br>☐N [0]
9	☐U5<br>☐ 5+<br>☐ NR	☐Y [1]<br>☐N [0]	☐Pos<br>☐Neg<br>☐NR	☐Mal<br>☐Not Mal<br>☐ NR	☐Y [1]<br>☐N [0]<br>☐NR [0]	☐ACT<br>☐Artes. Inj<br>☐Other<br>☐ NR	☐Y [1]<br>☐N [0]	☐Y<br>☐N	☐Y<br>☐N	☐Y<br>☐N	☐Pos<br>☐Neg<br>☐NF	☐Y<br>☐N<br>☐NF	☐Y [1]<br>☐N [0]
10	☐U5<br>☐ 5+<br>☐ NR	☐Y [1]<br>☐N [0]	☐Pos<br>☐Neg<br>☐NR	☐Mal<br>☐Not Mal<br>☐ NR	☐Y [1]<br>☐N [0]<br>☐NR [0]	☐ACT<br>☐Artes. Inj<br>☐Other<br>☐ NR	☐Y [1]<br>☐N [0]	☐Y<br>☐N	☐Y<br>☐N	☐Y<br>☐N	☐Pos<br>☐Neg<br>☐NF	☐Y<br>☐N<br>☐NF	☐Y [1]<br>☐N [0]
		**[__]/10**			**[__]/10**		**[__]/10**						**[__]/10**
		**____%**			**____%**		**____%**						**____%**
		**G.4.1 Malaria testing vs. febrile cases**			**G.4.2 Diagnosis matches test result**		**G.4.3 ACT treatment matches malaria diagnosis [OPD]**			**G.4.4 ACT treatment matches malaria test result [Test & Dispensing Register]**			
		**SCORE: Section G. Sample Register Review**								**[G4.1+G4.2+G4.3+G4.4] = _______/4 =_____%**			

Score Summary. OPD-DQA Overall Score

Instructions: Calculate Total OPD-DQA Score by averaging section Scores

**G.1. OPD-DQA Reporting Performance**	**_______%**	**G.3 OPD-DQA Consistency Check**	**_______%**
**G.2.OPD-DQA Readiness**	**_______%**	**G.4 OPD Sample Register Review**	**_______%**

Section H. Quality Improvement Plan

Instructions: Review the information from today's visit and the previous action plans to identify the top priority issues to address at this OPD site. Prepare the quality improvement plan together with the health facility providers, using the space below to record up to five action plans. Note: high-performing OPD sites may not require any quality improvement plans.

Which section of the checklist does the improvement plan correspond with?	Score (paper checklist only)	Describe the impediment	What immediate action was done?	What is the action plan?	Who is the person responsible?	What is the time frame?
☐OPD Site Readiness<br>☐OPD Observations<br>☐Patient Satisfaction<br>☐OPD-DQA	____________%					
☐OPD Site Readiness<br>☐OPD Observations<br>☐Patient Satisfaction<br>☐OPD-DQA	____________%					
☐OPD Site Readiness<br>☐OPD Observations<br>☐Patient Satisfaction<br>☐OPD-DQA	____________%					

	**Supervisor**	**Supervisee**
**Name**		
**Signature**		
**Date**		

**Date and end time of the Supervision visit**	|dd|mm|yyyy|	|hh|mm| ☐ AM/ ☐ PM

Sample Register Review Sheet

Instructions: Use this sheet to help with the selection and tracking of patients for Section H. OPD Register Review. Select the first ten patients from the start of the past month OPD register who have the following criteria: a) were prescribed antimalarial and/or antibiotic, b) tested for malaria, or c) with malaria diagnosis.

Once the register review has been completed, destroy this sheet by ripping up and discard the paper with names for confidentiality.

#	Name	ID	Age	Sex
1				
2				
3				
4				
5				
6				
7				
8				
9				
10				

NATIONAL
MALARIA
CONTROL
PROGRAM

USAID
FROM THE AMERICAN PEOPLE

CDC

U.S. President's Malaria Initiative

Swiss TPH
Swiss Tropical and Public Health Institute
Schweizerisches Tropen- und Public Health-Institut

The Global Fund
To Fight AIDS, Tuberculosis and Malaria
