# Supplementary figures and images for: The quality of malaria case management in different transmission settings in Tanzania mainland, 2017–2018
Source: PLOS Glob Public Health. 2023 Aug 21;3(8):e0002318. doi: 10.1371/journal.pgph.0002318 (PMC10441786; doi:10.1371/journal.pgph.0002318)

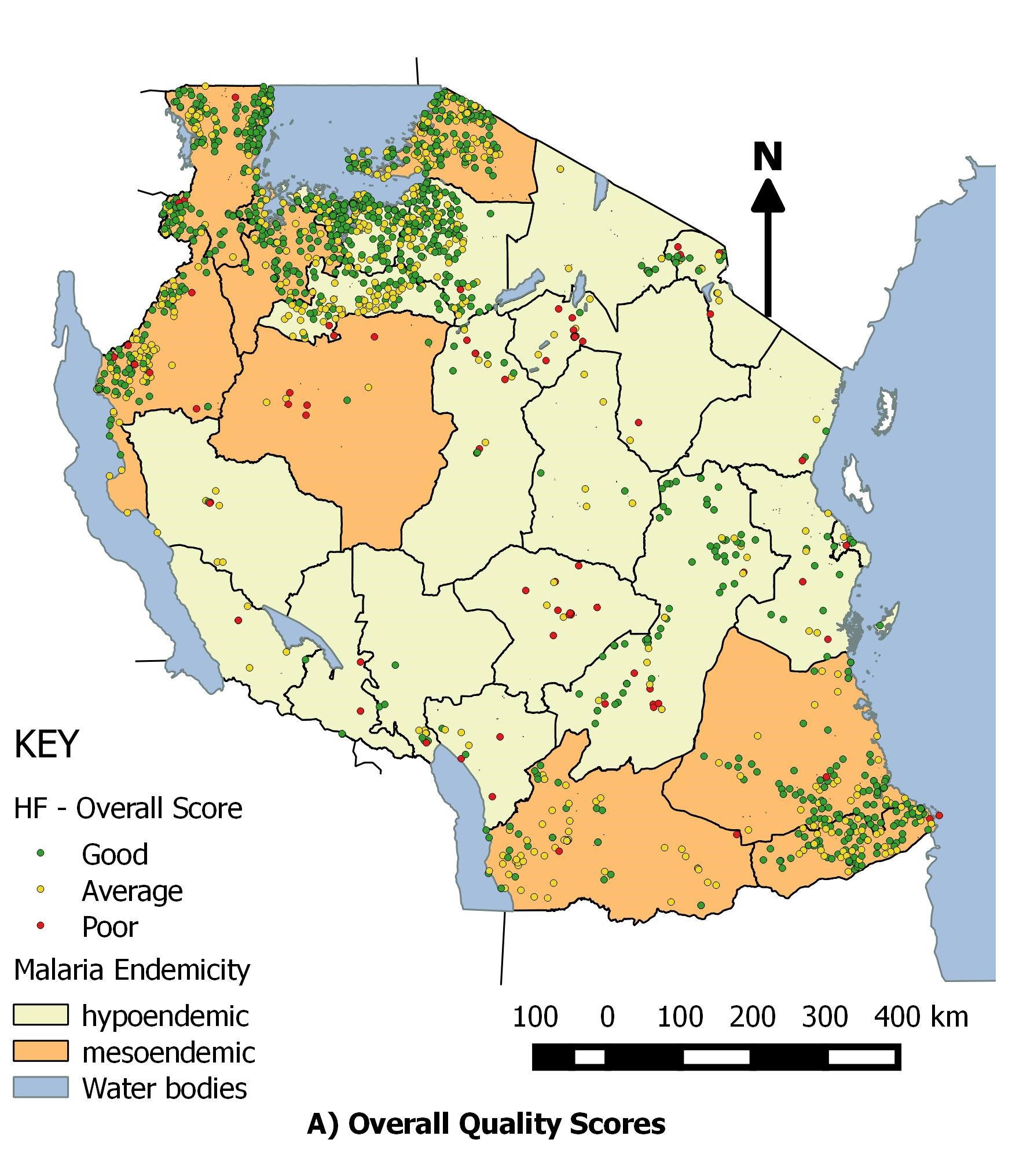

Supplement: S1 Fig — (TIF) [file pgph.0002318.s002.tif]

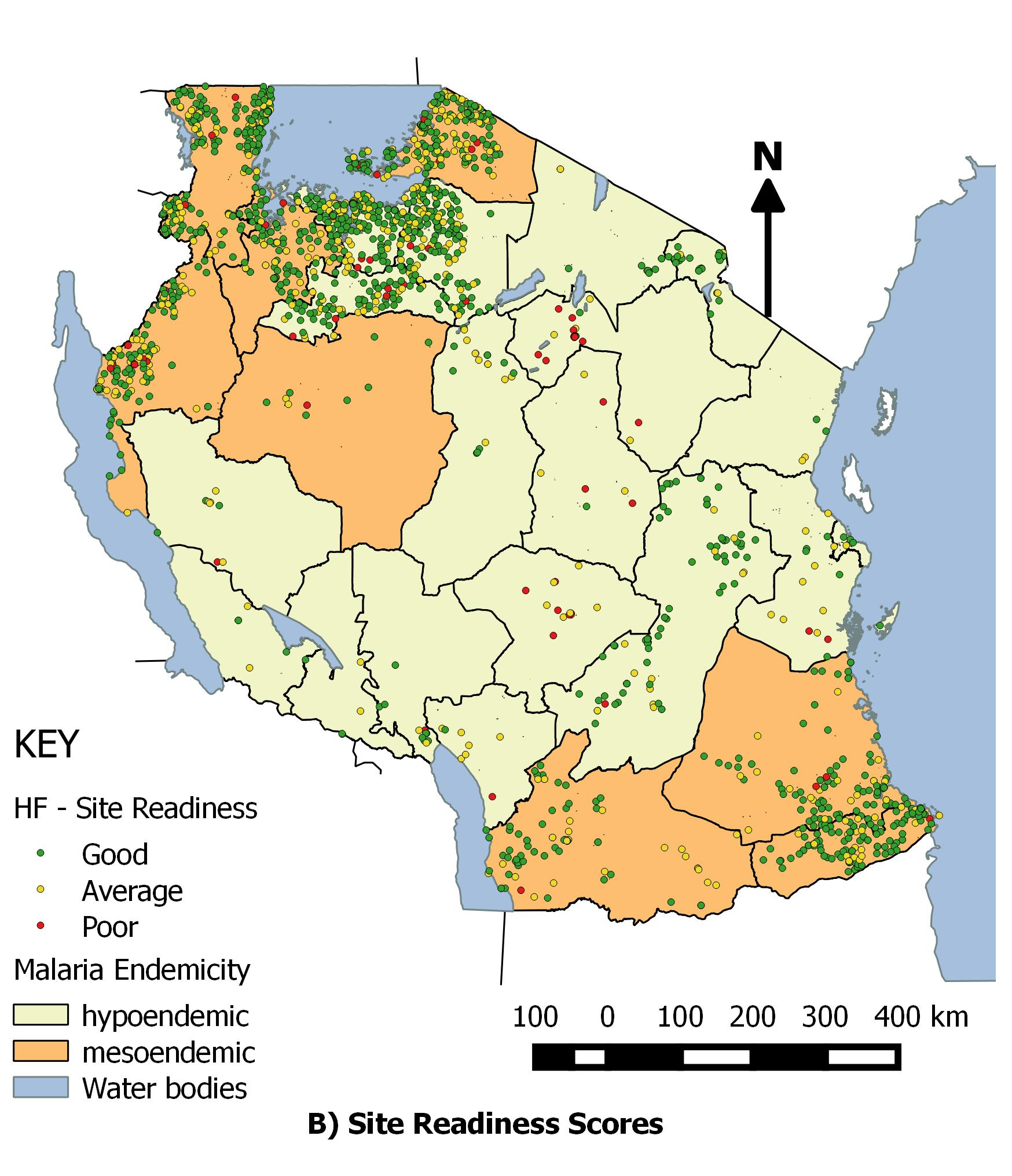

Supplement: S2 Fig — (TIF) [file pgph.0002318.s003.tif]

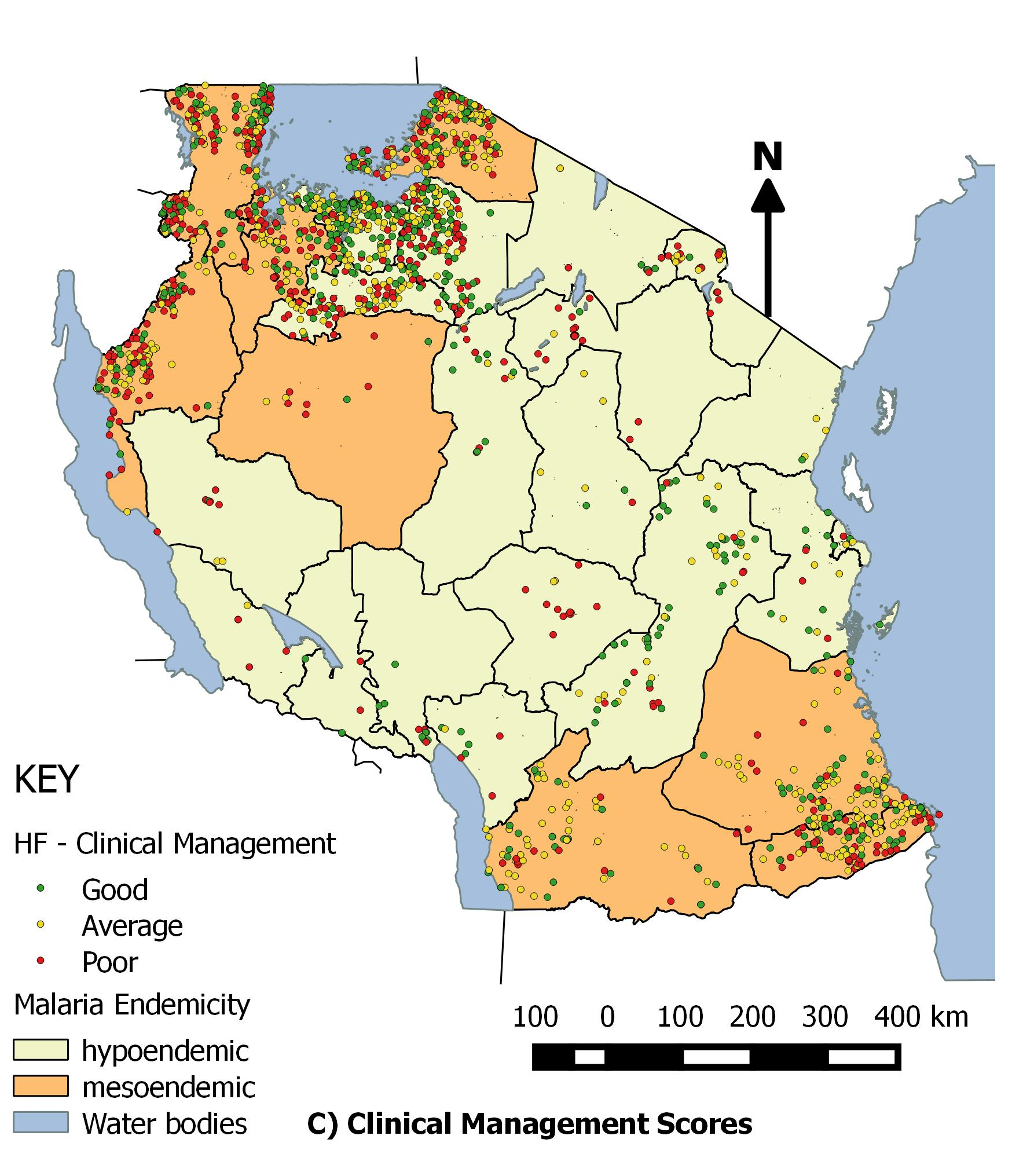

Supplement: S3 Fig — (TIF) [file pgph.0002318.s004.tif]

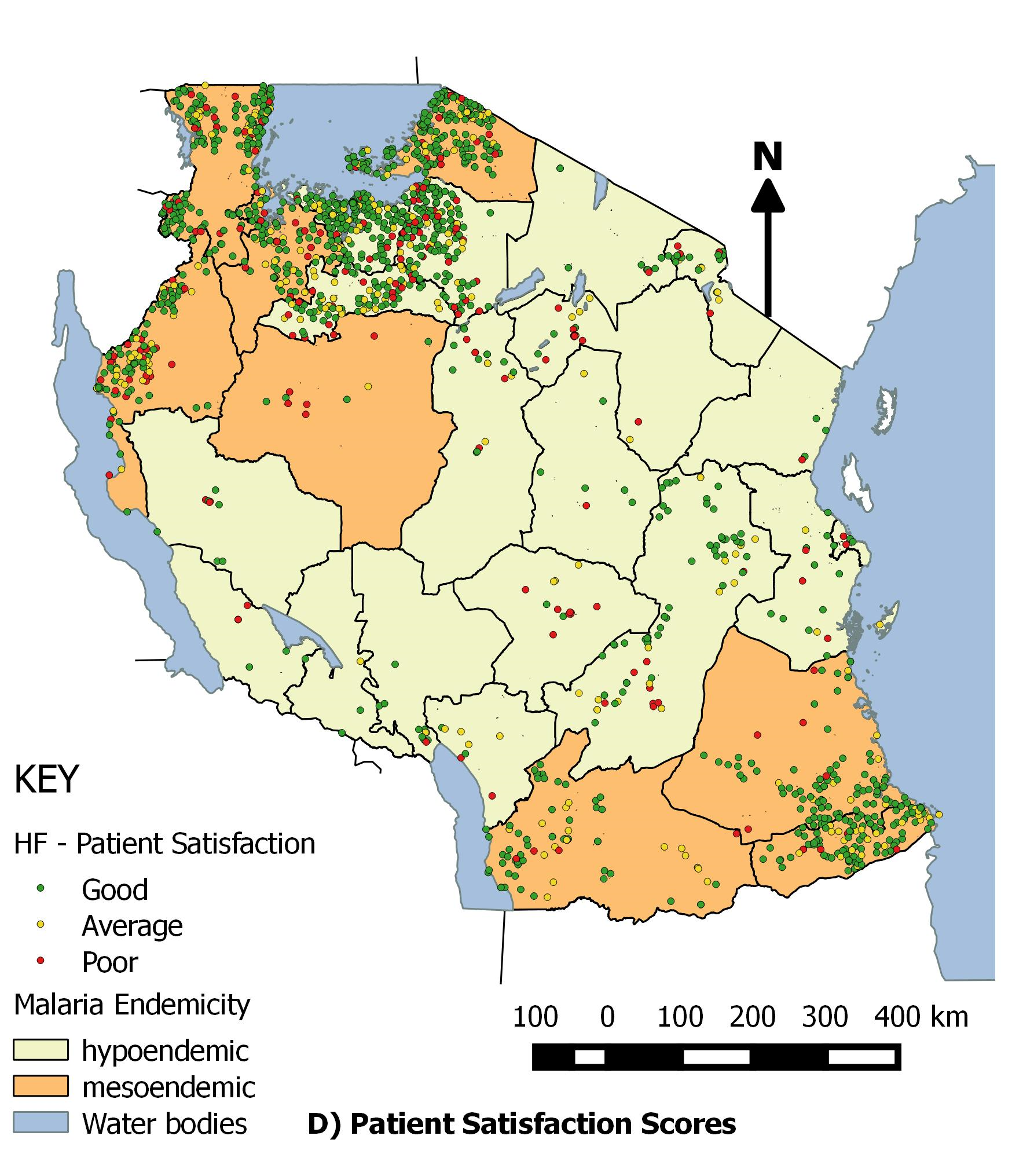

Supplement: S4 Fig — (TIF) [file pgph.0002318.s005.tif]
