# Supplementary material for: The quality of malaria case management in different transmission settings in Tanzania mainland, 2017–2018
Source: PLOS Glob Public Health. 2023 Aug 21;3(8):e0002318. doi: 10.1371/journal.pgph.0002318 (PMC10441786; doi:10.1371/journal.pgph.0002318)
Supplement: S1 Table — (DOCX) [file pgph.0002318.s006.docx]

**S1 Table: Health Facility MSDQI OPD Coverage by Region**

| **Region** | **Facilities studied (MSDQI OPD supervision conducted)**  **n (%)** | **Total Facilities operating**  **n** |
| --- | --- | --- |
| Arusha | 12 (3.0) | 400 |
| Dar es Salaam | 4 (0.5) | 803 |
| Dodoma | 7 (1.7) | 412 |
| Geita | 126 (64.9) | 194 |
| Iringa | 13 (4.9) | 265 |
| Kagera | 214 (66.0) | 324 |
| Katavi | 9 (11.8) | 76 |
| Kigoma | 135 (50.0) | 270 |
| Kilimanjaro | 28 (7.0) | 400 |
| Lindi | 108 (47.6) | 227 |
| Manyara | 21 (9.7) | 216 |
| Mara | 171 (57.4) | 298 |
| Mbeya | 11 (3.2) | 342 |
| Morogoro | 81 (20.8) | 389 |
| Mtwara | 135 (55.6) | 243 |
| Mwanza | 206 (50.2) | 410 |
| Njombe | 8 (3.0) | 268 |
| Pwani | 17 (5.3) | 320 |
| Rukwa | 5 (2.3) | 215 |
| Ruvuma | 89 (30.0) | 297 |
| Shinyanga | 93 (42.7) | 218 |
| Simiyu | 182 (74.3) | 245 |
| Singida | 19 (8.2) | 232 |
| Songwe | 6 (2.7) | 219 |
| Tabora | 9 (2.8) | 327 |
| Tanga | 4 (0.9) | 423 |
