# Supplementary material for: The quality of malaria case management in different transmission settings in Tanzania mainland, 2017–2018
Source: PLOS Glob Public Health. 2023 Aug 21;3(8):e0002318. doi: 10.1371/journal.pgph.0002318 (PMC10441786; doi:10.1371/journal.pgph.0002318)
Supplement: S2 Table — (DOCX) [file pgph.0002318.s007.docx]

**S2 Table: Distribution of all operating health facilities in Tanzania mainland by Endemicity**

| **Variables** | **Categories** | **Mesoendemic**  **(n= 2179)**  **n (%)** | **Hypoendemic**  **(n=5854)**  **n (%)** | **Total**  **(n= 8033)**  **n (%)** | **P-value** |
| --- | --- | --- | --- | --- | --- |
|  |  |  |  |  |  |
| Health facility level | Hospital | 71 (3.3) | 214 (3.7) | 285 (3.5) | 0.097 |
|  | Health Centre | 224 (10.3) | 592 (10.1) | 816 (10.2) |  |
|  | Dispensary | 1872 (85.9) | 4982 (85.1) | 6854 (85.3) |  |
|  | Others | 12 (0.5) | 66 (1.1) | 78 (1.0) |  |
|  |  |  |  |  |  |
| Health facility managing authority | Public | 1770 (81.2) | 3905 (66.7) | 5675 (70.7) | <0.001 |
|  | NGO/FBO | 212 (9.7) | 818 (14.0) | 1030 (12.8) |  |
|  | Private for profit | 197 (9.1) | 1131 (19.3) | 1328 (16.5) |  |
|  |  |  |  |  |  |
| Health facility location | Urban | 403 (18.5) | 1741 (29.7) | 2144 (26.7) | <0.001 |
|  | Rural | 1776 (81.5) | 4113 (70.3) | 5889 (73.3) |  |
|  |  |  |  |  |  |
| Partner support | Yes | 1853 (85.0) | 1263 (21.6) | 3116 (38.8) | <0.001 |
|  | No | 326 (15.0) | 4591 (78.4) | 4917 (61.2) |  |
| NGO=Non-Governmental Organization; FBO=Faith-Based Organization | | | | | |
